# Chemical composition of the lipophilic compounds from the rind and pith of papyrus (*Cyperus papyrus* L.) stems

**DOI:** 10.3389/fpls.2022.1097866

**Published:** 2022-12-22

**Authors:** Mario J. Rosado, Gisela Marques, Jorge Rencoret, Ana Gutiérrez, Florian Bausch, Thomas Rosenau, Antje Potthast, José C. del Río

**Affiliations:** ^1^ Instituto de Recursos Naturales y Agrobiología de Sevilla, CSIC, Seville, Spain; ^2^ Institute of Chemistry of Renewable Resources, University of Natural Resources and Life Sciences Vienna (BOKU), Tulln, Austria

**Keywords:** papyrus, phytadiene, fatty acids, phytol, ferulic acid, steroids, tocopherols

## Abstract

Papyrus (*Cyperus papyrus* L.) is a sedge plant with a high rate of biomass productivity that represents an interesting raw material to produce chemicals, materials and fuels, which are currently still obtained from fossil resources, in the context of a lignocellulosic biorefinery. In this work, the content and chemical composition of the lipids present in papyrus stems were thoroughly studied. For this, the papyrus stems were separated into the rind and the pith. The lipid content accounted for 4.1% in the rind and 4.9% in the pith (based on dry matter). The main compounds identified in both parts of the papyrus stem were hydrocarbons, *n*-fatty acids, 2-hydroxyfatty acids, alcohols, alkylamides, mono- and diglycerides, steroids (sterols, ketones, hydrocarbons, esters and glycosides), tocopherols, tocopherol esters, phytol, phytol esters, alkyl ferulates, ω-carboxyalkyl ferulates (and their monoglycerides), and acylglycerol glycosides. The rind presented a predominance of *n*-fatty acids (6790 mg/kg; that represented 28.6% of all identified compounds), steroid compounds (6255 mg/kg; 26.3%), phytol and phytol esters (4985 mg/kg; 21.0%), and isoprenoid hydrocarbons, namely phytadiene and squalene (2660 mg/kg; 11.2%), while the most abundant lipids in the pith were steroids (8600 mg/kg; 44.4% of all identified compounds) and fatty acids (6245 mg/kg; 32.2%). Due to the great diversity and significant abundance of the compounds identified in papyrus, it can be considered as a potential raw material for biorefineries to obtain valuable phytochemicals of interest to various industrial sectors.

## Introduction

Papyrus (*Cyperus papyrus* L.) is a sedge from the Cyperaceae family native to wetlands of central, eastern and southern Africa. Papyrus has traditionally been used to make fences, roofs and mats, as well as fiberboard and briquettes (Muthuri et al., 1989). Additionally, ancient civilizations used the pith of papyrus stems to make papyrus sheets, which had been an important writing surface (Nicholson and Shaw, 2000; Bausch et al., 2022). But more importantly, papyrus shows a strikingly high biomass productivity rate, with an aerial net primary production reaching up to 136.4 t DM ha^-1^ yr^-1^ (Jones et al., 2018; Rosado et al., 2021). The high productivity rate, and the fact that the plant also grows throughout the year, make papyrus an interesting source of biomass for the production of chemicals, materials and fuels in the context of a lignocellulosic biorefinery.

Full and efficient utilization of any plant biomass requires detailed knowledge of its composition in order to envisage and design optimized pathways for deconstruction and utilization. For this purpose, the content and composition of the main components of papyrus stems have already been addressed (Rosado et al., 2021), and the composition and structure of the lignins in the two differentiated anatomical parts of the stems (rind and pith) have already been investigated in detail (Rosado et al., 2021; Rencoret et al., 2022). Although carbohydrates (cellulose and hemicelluloses) and lignin are the main biomass components of interest in lignocellulosic biorefineries (Ragauskas et al., 2006; Kumar and Verma, 2021), other minor plant constituents, such as lipidic extractives, are generally not being considered, although they undoubtedly have great potential as a source of valuable lipids of industrial interest (Marques et al., 2020; Verkasalo et al., 2021; Rosado et al., 2022). As previously shown, papyrus stems presented a significant content of lipophilic extractives, 4.1% in the rind and 4.9% in the pith (Rosado et al., 2021), which makes it a potential source for obtaining valuable lipids. Plant lipids include a wide range of compounds, such as hydrocarbons, fatty acids, fatty alcohols, steroids, triterpenoids, or long chain esters, among others (del Río et al., 2016; Marques et al., 2020; Rosado et al., 2022). The diverse chemical structure of plant lipids is generally of great potential in a wide range of industrial applications, such as in cosmetic, pharmaceutical, nutraceutical, or chemical industries (Hernández, 2005; Metzger and Bornscheuer, 2006; Tao, 2007; Carciochi et al., 2017; Attard et al., 2018).

However, and despite its interest, there is a lack of studies addressing the detailed composition of the lipids of papyrus stems. A previous paper only reported the lipid composition in papyrus tubers but not in the stems (Hassanein et al., 2011) while other studies reported the composition of the essential oils in the aerial parts (Sonwa, 2000; Hassanein et al., 2014). Therefore, in this work, the lipid compounds present in acetone extracts from the rind and pith of papyrus stems were thoroughly analyzed by gas chromatography-mass spectrometry (GC-MS) using a medium-length, high-temperature capillary column, with thin films, according to the method developed by our group that allowed the elution and identification of a wide range of components, from low molecular weight fatty acids to high molecular weight sterol esters, sterol glycosides, or long chain ester waxes (Gutiérrez et al., 1998; Gutiérrez et al., 2004). The results presented here will greatly improve our understanding of the lipidic compounds present in this interesting plant, and will help to maximize its complete exploitation.

## Materials and methods

### Samples

Papyrus (*Cyperus papyrus* L.) plants were grown in an open field in Qaramos, Egypt. The papyrus samples studied here were collected in March 2020, although harvesting can be done throughout the year. Papyrus plants were air-dried immediately after collection, and the pith and the rind of the stems of two plants were separated manually using a cutter and mixed. The separated rind and pith fractions were milled using a knife mill to successively pass through 2 and 1 mm sieves. The samples were then extracted with acetone in a Soxhlet for 12 hours. The acetone extracts were brought to dryness using a rotary evaporator and the content determined gravimetrically, which accounted for 4.1% ± 0.1 in the rind and 4.9% ± 0.4 in the pith (on a dry basis). The determinations were performed in triplicate.

### GC-MS analysis of lipids

The acetone extracts were analyzed by GC−MS both underivatized, and after derivatization with *N,O*-bis(trimethylsilyl)trifluoroacetamide (BSTFA) to form their trimethylsilyl (TMS) ether derivatives. The analyses were performed on a Shimadzu QP 2010 Ultra GC-MS system (Kyoto, Japan) using a fused-silica DB-5HT capillary column (15 m length x 0.25 mm i.d., 0.1 μm film thickness) from J&W Scientific (Folsom, CA, USA). The oven was heated from 120°C (1 min) to 380°C at 10°C min^−1^ and held for 5 min. The injection was performed at 300°C and the transfer line was kept at 300°C. Helium was used as the carrier gas at a rate of 1 mL min^−1^. The different compounds were identified by comparison of their mass spectra with those in the NIST library, by comparison with literature and, when possible, by comparison with authentic standards. Quantification was performed by using a mixture of authentic standards (tetracosane, palmitic acid, linoleic acid, stigmasterol, sitosterol, cholesteryl linoleate, sitosteryl 3-*O*-β-d-glucopyranoside, sitosteryl (6*′*-*O*-palmitoyl) 3-*O*-β-d-glucopyranoside, 1-monopalmitin, 1,3-dipalmitin, tripalmitin) in a concentration range between 0.1 and 1.0 mg/mL. Quantification was given as the mean of three replicates of the same sample.

## Results and discussion

### Lipid composition of the rind and pith of papyrus stems

**Figure 1:**
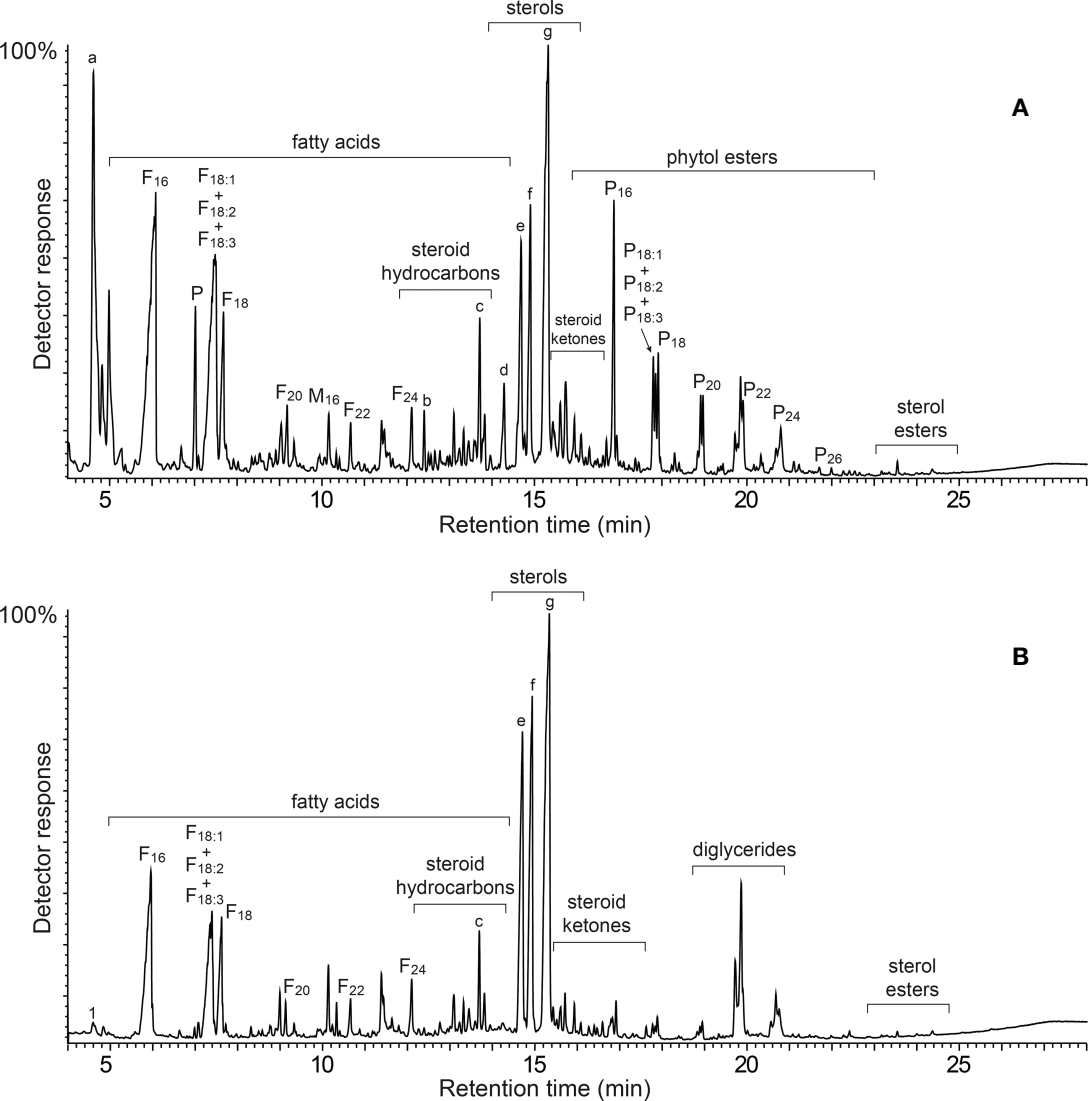
GC-MS chromatograms of the underivatized lipid extracts from **(A)** the rind, and **(B)** the pith of papyrus stems. F(n), *n*-fatty acid series; M(n), monoglycerides; P, phytol; P(n), phytol esters series. Labels for selected compounds are: a, phytadiene; b, squalene; c, stigmasta-3,5,22-triene; d, α-tocopherol; e, campesterol; f, stigmasterol; g, sitosterol.

**Figure 2:**
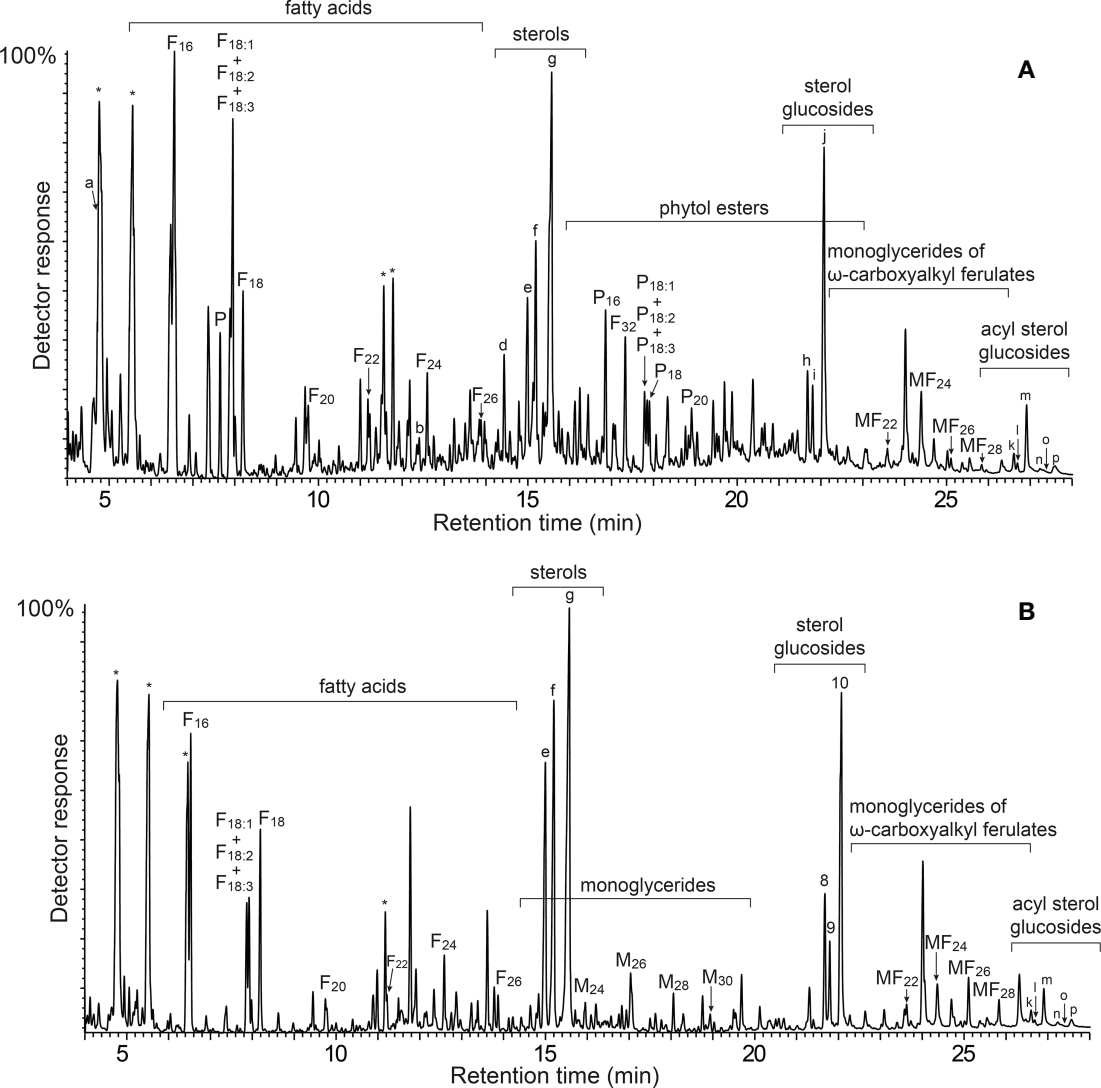
GC-MS chromatograms of the TMS-ether derivatives of the lipid extracts from **(A)** the rind, and **(B)** the pith of papyrus stems. F(n), *n*-fatty acid series; M(n), monoglycerides; P, phytol; P(n), phytol esters series; MF(n), monoglycerides of ω-carboxyalkyl ferulates. Labels for selected compounds are: a, phytadiene; b, squalene; c, stigmasta-3,5,22-triene; d, α-tocopherol; e, campesterol; f, stigmasterol; g, sitosterol; h, campesteryl 3-*O*-β-d-glucopyranoside; i, stigmasteryl 3-*O*-β-d-glucopyranoside; j, sistosteryl 3-*O*-β-d-glucopyranoside; k, campesteryl (6′-*O*-palmitoyl)-3-*O*-β-d-glucopyranoside; l, stigmasteryl (6′-*O*-palmitoyl)-3-*O*-β-d-glucopyranoside; m, sistosteryl (6′-*O*-palmitoyl)-3-*O*-β-d-glucopyranoside; n, campesteryl (6′-*O*-stearoyl/oleyl/linoleyl/linolenyl)-3-*O*-β-d-glucopyranoside; o, stigmasteryl (6′-*O*-stearoyl/oleyl/linoleyl/linolenyl)-3-*O*-β-d-glucopyranoside; p, sistosteryl (6′-*O*-stearoyl/oleyl/linoleyl/linolenyl)-3-*O*-β-d-glucopyranoside. * Unknown compounds.

The acetone extracts of the rind (4.1%) and pith (4.9%) of papyrus stems were redissolved in chloroform and subsequently analyzed by GC-MS according to the method previously described (Gutiérrez et al., 1998; Gutiérrez et al., 2004) that used medium-length high temperature capillary columns, that allowed the identification of lipid compounds from low molecular weight fatty acids to high molecular weight compounds, such as sterol esters and sterol glycosides, among others. For a complete and more convenient identification of the compounds, the extracts were analyzed both underivatized, and as their trimetylsilyl (TMS) ether derivatives. The chromatograms of the lipidic extracts of the rind and pith of papyrus stems (underivatized and as their TMS-ether derivatives) are shown in **Figures 1**, **2**.

**Figure 3:**
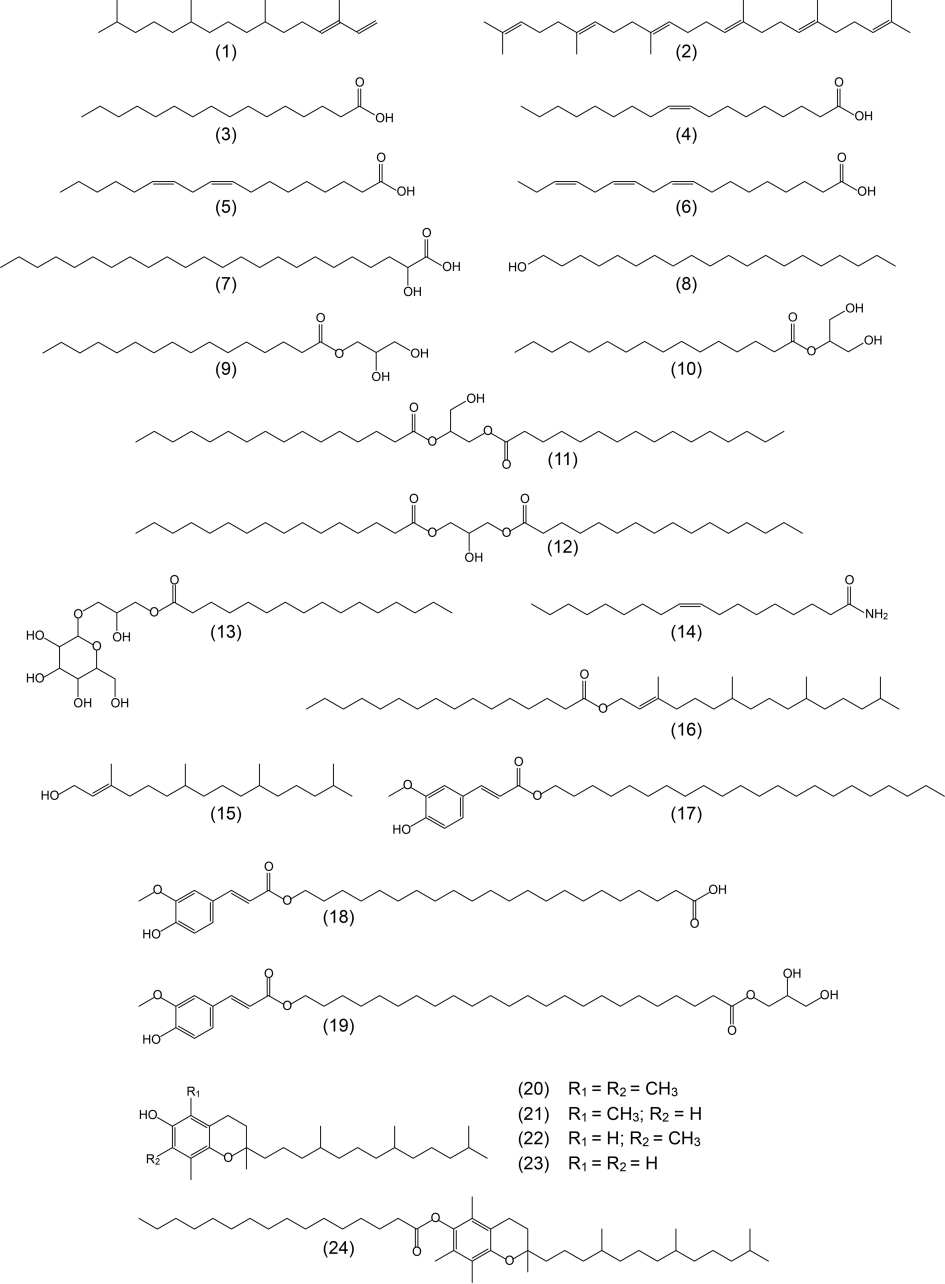
Structures representative of the main aliphatic compounds, ferulic acid derivatives, and tocopherols identified in the acetone extracts of the rind and pith of papyrus stems and referred in the text. 1: phytadiene; 2: squalene; 3: *n*-hexadecanoic (palmitic) acid; 4: *cis*-octadec-9-enoic (oleic) acid; 5: *cis*,*cis*-octadeca-9,12-dienoic (linoleic) acid; 6: *cis*,*cis,cis*-octadeca-9,12,15-trienoic (linolenic) acid; 7: 2-hydroxytetracosanoic acid; 8: *n*-eicosanol; 9: 1-monohexadecanoylglycerol (1-monopalmitin); 10: 2-monohexadecanoylglycerol (2-monopalmitin); 11: 1,2-dipalmitin; 12: 1,3-dipalmitin; 13: lyso-monogalactosyl-monopalmitin; 14: oleic amide; 15: phytol; 16: phytyl hexadecanoate; 17: docosanyl ferulate; 18: feruloyloxydocosanoic acid; 19: 1-mono-*trans*-feruloyloxytetracosanoylglycerol; 20: α-tocopherol; 21: β-tocopherol; 22: γ-tocopherol; 23: δ-tocopherol; 24: α-tocopheryl hexadecanoate.

**Figure 4:**
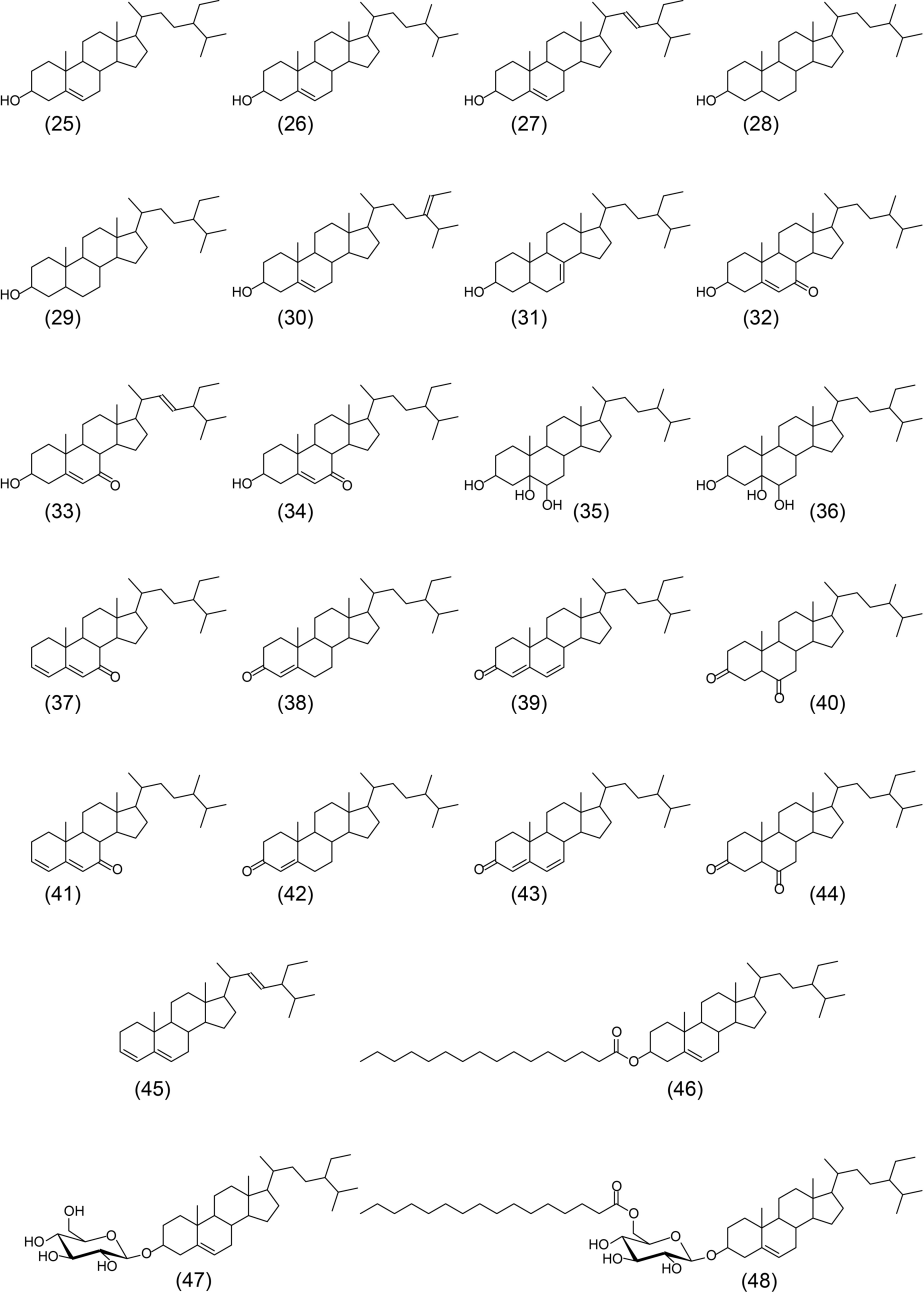
Structures of the main steroid compounds identified in the acetone extracts of the rind and pith of papyrus stems and referred in the text. 25: sitosterol; 26: campesterol; 27: stigmasterol; 28: campestanol; 29: stigmastanol; 30: Δ^5^-avenasterol; 31: Δ^7^-stigmastenol; 32: 7-oxocampesterol; 33: 7-oxo-stigmasterol; 34: 7-oxositosterol; 35: ergostane-3,5,6-triol; 36: sitostane-3,5,6-triol; 37: stigmasta-3,5-dien-7-one; 38: stigmast-4-en-3-one; 39: stigmasta-4,6-dien-3-one; 40: stigmastane-3,6-dione; 41: ergosta-3,5-dien-7-one; 42: ergost-4-en-3-one; 43: ergosta-4,6-dien-3-one; 44: ergostane-3,6-dione; 45: stigmasta-3,5,22-triene; 46: sitosteryl hexadecanoate; 47: sitosteryl 3-*O*-β-d-glucopyranoside; 48: sitosteryl (6’-*O*-palmitoyl)-3-*O*-β-d-glucopyranoside.

**Figure 5:**
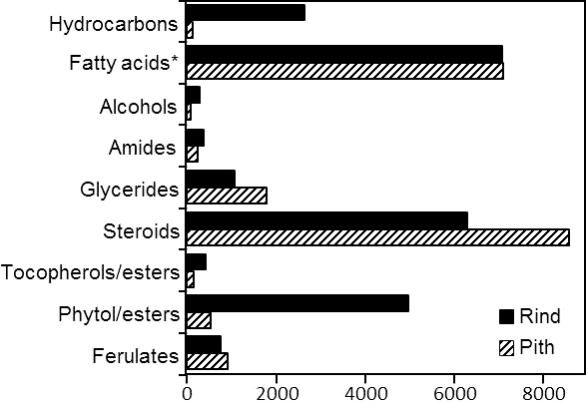
Abundance (mg/kg, dry-basis) of the different classes of lipid compounds identified in the acetone extracts of rind and pith of papyrus stems. *The abundance of fatty acids also includes the 2-hydroxyfatty acids.

A wide range of lipidic compounds were identified, including hydrocarbons, fatty acids, 2-hydroxyfatty acids, fatty alcohols, phytol and phytol esters, alkylamides, mono- and diglycerides, steroids (sterols, ketones, hydrocarbons, esters and glycosides), tocopherols and tocopheryl esters, alkyl ferulates, ω-carboxyalkyl ferulates (and their monoglycerides), and acylglycerol glycosides. The mass spectra of representative compounds are shown in **Figures S1–S11** in **Supplementary Material**. The identities and abundances (as mg/kg, dry-free basis) of all compounds identified in the rind and pith of papyrus stems are listed in **Table 1**. Representative structures of the different classes of lipids identified are shown in **Figure 3** (for aliphatic compounds, ferulic acid derivatives, tocopherols and their ester) and in **Figure 4** (for steroid compounds). The abundances of the different lipid classes in both parts of the papyrus stems are indicated in the histogram of **Figure 5**, which clearly evidences their completely different composition. The rind contained predominantly free fatty acids (6790 mg/kg; 28.6% of all identified compounds), steroid compounds (6255 mg/kg; 26.3%), phytol and phytol esters (4985 mg/kg; 21.0%), and isoprenoid hydrocarbons, namely phytadiene and squalene (2660 mg/kg; 11.2%). On the other hand, the most abundant lipids in the pith were steroid compounds (8600 mg/kg; 44.4% of all identified compounds) and free fatty acids (6245 mg/kg; 32.2%).

**Table 1:** Composition and abundance (mg/kg, dry-basis) of the lipid compounds identified in the acetone extracts of the rind and pith of papyrus stems (values are the mean of three technical replicates; values in bold correspond to the total amounts of each group of compounds and the standard deviation).

Compounds	Rind	Pith	Compounds	Rind	Pith
**Hydrocarbons**	**2660 ± 150**	**100 ± 10**	**Steroid hydrocarbons**	**710 ± 80**	**640 ± 50**
phytadiene	2340	30	ergosta-3,5,22-triene	140	135
squalene	320	70	ergosta-3,5-diene	60	40
			stigmasta-3,5,22-triene	430	340
***n*-Fatty acids**	**6790 ± 500**	**6245 ± 400**	stigmasta-3,5-diene	80	125
*n*-tetradecanoic acid	90	155			
*n*-pentadecanoic acid	50	65	**Sterol esters**	**365 ± 60**	**200 ± 20**
*n*-hexadecanoic acid	2060	2210	campesteryl dodecanoate	15	5
*n*-heptadecanoic acid	95	45	stigmasteryl dodecanoate	8	5
*cis,cis,cis*-octadeca-9,12,15-trienoic acid	500	75	sitosteryl dodecanoate	26	8
*cis,cis*-octadeca-9,12-dienoic acid	858	610	campesteryl tetradecanoate	22	5
*cis*-octadec-9-enoic acid	825	790	stigmasteryl tetradecanoate	8	5
*n*-octadecanoic acid	550	1075	sitosteryl tetradecanoate	30	10
*n*-nonadecanoic acid	22	40	campesteryl hexadecanoate	44	20
*n*-eicosanoic acid	165	145	stigmasteryl hexadecanoate	16	10
*n*-heneicosanoic acid	25	25	sitosteryl hexadecanoate	120	45
*n*-docosanoic acid	143	145	campesteryl octadecanoate/oleate/linoleate	15	27
*n*-tricosanoic acid	94	115	stigmasteryl octadecanoate/oleate/linoleate	11	15
*n*-tetracosanoic acid	220	330	sitosteryl octadecanoate/oleate/linoleate	50	45
*n*-pentacosanoic acid	90	105			
*n*-hexacosanoic acid	120	150	**Sterol glycosides**	**1780 ± 50**	**2140 ± 200**
*n*-heptacosanoic acid	15	15	campesteryl 3-*O*-β-d-glucopyranoside	210	400
*n*-octacosanoic acid	170	110	stigmasteryl 3-*O*-β-d-glucopyranoside	140	210
*n*-nonacosanoic acid	5	5	sitosteryl 3-*O*-β-d-glucopyranoside	990	1210
*n*-triacontanoic acid	190	20	7-oxo-campesteryl 3-*O*-β-d-glucopyranoside	25	25
*n*-hentriacontanoic acid	20	5	7-oxo-stigmasteryl 3-*O*-β-d-glucopyranoside	15	20
*n*-dotriacontanoic acid	343	10	7-oxo-sitosteryl 3-*O*-β-d-glucopyranoside	40	40
*n*-tritriacontanoic acid	10	tr.	campesteryl (6′-*O*-palmitoyl)-3-*O*-β-d-glucopyranoside	50	50
*n*-tetratriacontanoic acid	100	tr.	stigmasteryl (6′-*O*-palmitoyl)-3-*O*-β-d-glucopyranoside	25	20
*n*-pentatriacontanoic acid	5	tr.	sitosteryl (6′-*O*-palmitoyl)-3-*O*-β-d-glucopyranoside	200	110
*n*-hexatriacontanoic acid	25	tr.	campesteryl (6′-*O*-stearoyl/oleyl/linoleyl/linolenyl)-3-*O*-β-d-glucopyranoside	25	20
			stigmasteryl (6′-*O*-stearoyl/oleyl/linoleyl/linolenyl)-3-*O*-β-d-glucopyranoside	15	5
**2-Hydroxy fatty acids**	**270 ± 25**	**830 ± 30**	sitosteryl (6′-*O*-stearoyl/oleyl/linoleyl/linolenyl)-3-*O*-β-d-glucopyranoside	45	30
2-hydroxyeicosanoic acid	55	178			
2-hydroxyheneicosanoic acid	5	5	**Tocopherols**	**205 ± 20**	**45 ± 10**
2-hydroxydocosanoic acid	30	84	α-tocopherol	120	25
2-hydroxytricosanoic acid	20	26	β-tocopherol	10	5
2-hydroxytetracosanoic acid	110	375	γ-tocopherol	40	10
2-hydroxypentacosanoic acid	25	50	δ-tocopherol	35	5
2-hydroxyhexacosanoic acid	25	112			
			**Tocopherol esters**	**220 ± 20**	**95 ± 10**
***n*-Fatty alcohols**	**280 ± 25**	**65 ± 5**	α-tocopheryl dodecanoate	5	tr
*n*-eicosanol	66	15	α-tocopheryl tetradecanoate	35	15
*n*-heneicosanol	15	4	α-tocopheryl hexadecanoate	52	15
*n*-docosanol	62	15	α-tocopheryl oleate/linoleate	15	45
*n*-tricosanol	15	2	α-tocopheryl octadecanoate	30	tr
*n*-tetracosanol	18	5	α-tocopheryl eicosanoate	3	tr
*n*-pentacosanol	6	2	β-tocopheryl dodecanoate	3	tr
*n*-hexacosanol	30	8	β-tocopheryl tetradecanoate	8	5
*n*-heptacosanol	8	2	β-tocopheryl hexadecanoate	10	4
*n*-octacosanol	60	12	β-tocopheryl oleate/linoleate	3	1
			β-tocopheryl octadecanoate	8	tr
**Alkylamides**	**300 ± 25**	**175 ± 15**	β-tocopheryl eicosanoate	3	tr
oleic amide	185	175	γ-tocopheryl dodecanoate	3	tr
triacontanamide	25	nd	γ-tocopheryl tetradecanoate	8	5
dotriacontanamide	60	nd	γ-tocopheryl hexadecanoate	10	4
tetratriacontanamide	30	nd	γ-tocopheryl oleate/linoleate	3	1
			γ-tocopheryl octadecanoate	8	tr
**1-Monoglycerides**	**650 ± 80**	**830 ± 70**	γ-tocopheryl eicosanoate	3	tr
1-monohexadecanoylglycerol	135	88	δ-tocopheryl dodecanoate	2	tr
1-monoheptadecanoylglycerol	5	3	δ-tocopheryl tetradecanoate	2	tr
1-monooctadec-9,12,15-trienoylglycerol	75	6	δ-tocopheryl hexadecanoate	2	tr
1-monooctadec-9,12-dienoylglycerol	68	25	δ-tocopheryl oleate/linoleate	tr	tr
1-monooctadec-9-enoylglycerol	52	38	δ-tocopheryl octadecanoate	2	tr
1-monooctadecanoylglycerol	32	85	δ-tocopheryl eicosanoate	2	tr
1-monononadecanoylglycerol	3	6			
1-monoeicosanoylglycerol	40	78	**Phytol and phytol esters**	**4985 ± 200**	**540 ± 60**
1-monoheneicosanoylglycerol	3	4	phytol	260	20
1-monodocosanoylglycerol	22	28	phytyl dodecanoate	185	5
1-monotricosanoylglycerol	12	22	phytyl tridecanoate	25	tr
1-monotetracosanoylglycerol	38	75	phytyl tetradecanoate	320	20
1-monopentacosanoylglycerol	6	12	phytyl pentadecanoate	40	5
1-monohexacosanoylglycerol	40	174	phytyl hexadecanoate	1430	80
1-monoheptacosanoylglycerol	3	12	phytyl heptadecanoate	55	5
1-monooctacosanoylglycerol	48	140	phytyl octadeca-9,12,15-trienoate	358	40
1-monononacosanoylglycerol	3	4	phytyl octadeca-9,12-dienoate	600	60
1-monotriacontanoylglycerol	55	30	phytyl octadec-9-enoate	40	8
1-monohentriacontanoylglycerol	2	nd	phytyl octadecanoate	582	90
1-monodotriacontanoylglycerol	8	nd	phytyl nonadecanoate	44	10
			phytyl eicosanoate	420	55
**2-Monoglycerides**	**90 ± 5**	**120 ± 10**	phytyl heneicosanoate	38	10
2-monohexadecanoylglycerol	15	12	phytyl docosanoate	276	50
2-monotetracosanoylglycerol	16	20	phytyl tricosanoate	88	18
2-monohexacosanoylglycerol	22	42	phytyl tetracosanoate	164	40
2-monooctacosanoylglycerol	25	34	phytyl pentacosanoate	40	14
2-monotriacontanoylglycerol	12	12	phytyl hexacosanoate	20	10
**Diglycerides**	**270 ± 10**	**610 ± 30**	**Alkyl ferulates**	**90 ± 10**	**85 ± 5**
1,2-dipalmitin	60	70	*trans*-octadecyl ferulate	5	6
1,3-dipalmitin	75	90	*trans*-eicosanyl ferulate	15	8
1,2-palmitoylolein	60	120	*trans*-docosanyl ferulate	32	18
1,3-palmitoylolein	75	330	*trans*-tetracosanyl ferulate	8	10
			*trans*-hexacosanyl ferulate	20	28
**Acylglycerol glycosides**	**50 ± 5**	**200 ± 30**	*trans*-octacosanyl ferulate	10	15
lyso-monogalactosyl-monopalmitin	50	200			
			**ω-carboxyalkyl ferulates**	**340 ± 15**	**325 ± 20**
**Sterols**	**3080 ± 120**	**5420 ± 150**	*trans*-feruloyloxyeicosanoic acid	20	15
campestanol	96	220	*trans*-feruloyloxyheneicosanoic acid	10	10
campesterol	370	1040	*trans*-feruloyloxydocosanoic acid	90	70
stigmasterol	620	1350	*trans*-feruloyloxytricosanoic acid	10	10
sitosterol	1720	2280	*trans*-feruloyloxytetracosanoic acid	75	80
stigmastanol	160	200	*trans*-feruloyloxypentacosanoic acid	10	10
Δ^5^-avenasterol	16	100	*trans*-feruloyloxyhexacosanoic acid	65	80
Δ^7^-stigmastenol	10	30	*trans*-feruloyloxyheptacosanoic acid	15	10
7-oxo-campesterol	12	30	*trans*-feruloyloxyoctacosanoic acid	45	40
7-oxo-stigmasterol	20	40			
7-oxo-sitosterol	56	80	**Monoglycerides of ω-carboxyalkyl ferulates**	**325 ± 15**	**500 ± 10**
ergostane-3,5,6-triol	tr	20	1-mono*-trans*-feruloyloxyeicosanoylglycerol	10	15
sitostane-3,5,6-triol	tr	30	1-mono*-trans*-feruloyloxydocosanoylglycerol	85	70
			1-mono*-trans*-feruloyloxytetracosanoylglycerol	135	120
**Steroid ketones**	**320 ± 20**	**200 ± 20**	1-mono*-trans*-feruloyloxyhexacosanoylglycerol	55	190
ergosta-3,5-dien-7-one	10	16	1-mono*-trans*-feruloyloxyoctacosanoylglycerol	30	90
ergost-4-en-3-one	30	14	1-mono*-trans*-feruloyloxytriacontanoylglycerol	10	15
ergosta-4,6-dien-3-one	70	30			
stigmasta-3,5-dien-7-one	110	50			
stigmast-4-en-3-one	44	40			
stigmasta-4,6-dien-3-one	40	20			
ergostane-3,6-dione	6	10			
stigmastane-3,6-dione	10	20			

### Aliphatic compounds

**Figure 6:**
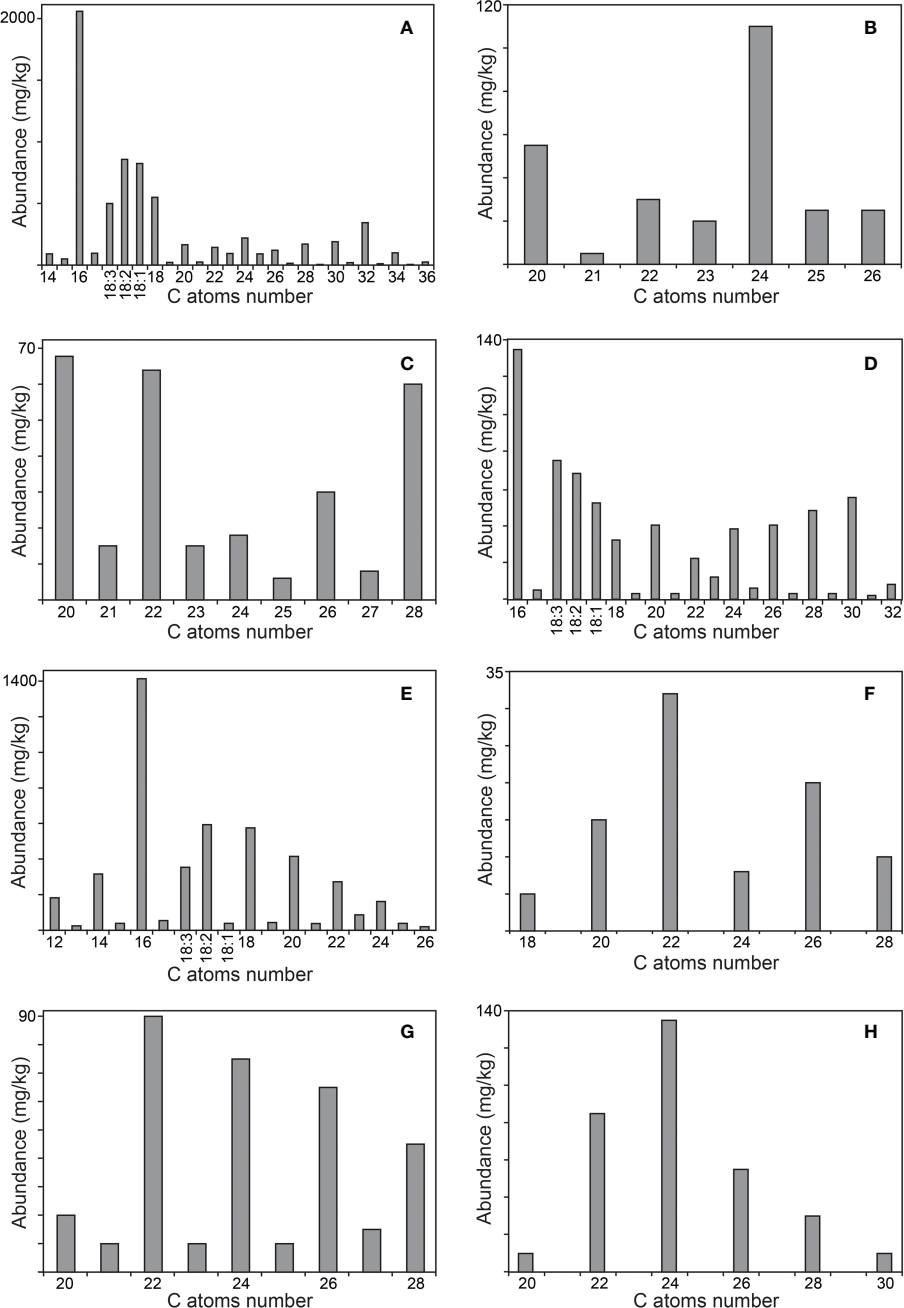
Distribution of the main aliphatic series identified in the acetone extracts of the rind of papyrus stems. **(A)** *n*-fatty acids; **(B)** 2-hydroxy fatty acids; **(C)** *n*-fatty alcohols; **(D)** 1-monoglycerides; **(E)** phytol esters; **(F)** alkyl ferulates; **(G)** ω-carboxyalkyl ferulates; **(H)** monoglycerides of ω-carboxyalkyl ferulates. The histograms are scaled up to the abundance of the major compound in the series.

**Figure 7:**
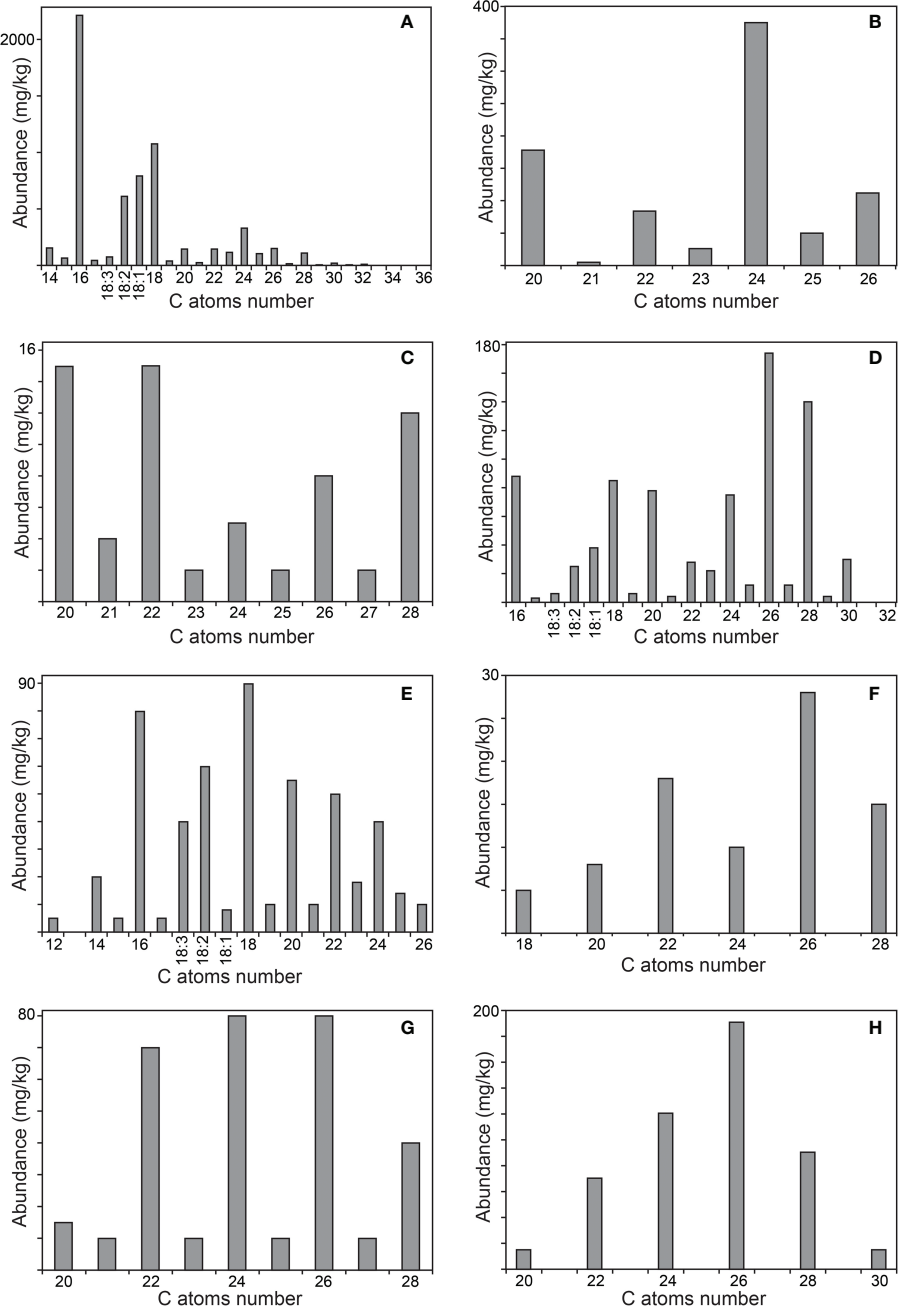
Distribution of the main aliphatic series identified in the acetone extracts of the pith of papyrus stems. **(A)** *n*-fatty acids; **(B)** 2-hydroxy fatty acids; **(C)** *n*-fatty alcohols; **(D)** 1-monoglycerides; **(E)** phytol esters; **(F)** alkyl ferulates; **(G)** ω-carboxyalkyl ferulates; **(H)** monoglycerides of ω-carboxyalkyl ferulates. The histograms are scaled up to the abundance of the major compound in the series.

The main classes of aliphatic compounds identified were hydrocarbons, free fatty acids, 2-hydroxyfatty acids, fatty alcohols, mono- and diglycerides, phytol and phytol esters. The distributions of the major series of aliphatic compounds present in the rind and pith of papyrus stems are shown in the histograms of **Figures 6**, **7**, respectively.

The only hydrocarbons identified were the isoprenoids phytadiene (1) and squalene (2) that were much more abundant in the rind than in the pith. Series of *n*-alkanes, which are common lipids in many plants (Coelho et al., 2007; del Río et al., 2013; Rosado et al., 2022), could not be identified in papyrus stems, although a series of alkanes was found in papyrus tubers (Hassanein et al., 2011). The most abundant hydrocarbon in the rind was phytadiene, which accounted for 2340 mg/kg, while squalene amounted up to 320 mg/kg. In the pith, these isoprenoid hydrocarbons were present in lesser amounts, with squalene (70 mg/kg) being more prominent than phytadiene (30 mg/kg).

*n*-Fatty acids were present in similar abundance in the rind (6790 mg/kg), and the pith (6245 mg/kg), in a series ranging from *n*-tetradecanoic acid (C_14_) to *n*-hexatriacontanoic acid (C_36_), with a strong predominance of even over odd carbon number homologues, as indicated in the histograms of **Figures 6A**, **7A**. The major compound in the series was hexadecanoic acid (palmitic acid, C_16_; 3) that accounted for 2060 mg/kg in the rind and 2210 mg/kg in the pith. Interestingly, significant amounts of high molecular weight fatty acids above C_30_ were found in the rind, but they were barely detected in the pith. Important amounts of the unsaturated oleic (C_18:1_; 4), linoleic (C_18:2_; 5) and linolenic (C_18:3_; 6) acids were also found in papyrus stems, with linolenic acid being particularly abundant in the rind (500 mg/kg), but present only in low amounts in the pith (75 mg/kg). The amounts of fatty acids identified in papyrus stems are among the highest values found in other lignocellulosic materials, and compare to the high amounts of fatty acids reported in maize fibers of 7590 mg/kg (Marques et al., 2020).

A series of 2-hydroxyfatty acids were also observed in the papyrus samples, accounting for 270 mg/kg in the rind and 830 mg/kg in the pith. The identification of these compounds was made based on the characteristic mass spectra of their TMS-ether derivatives, which exhibit a base peak fragment arising from the loss of the carboxyl group (as TMS-derivative), as shown in **Figure S1** for a representative member (2-hydroxytetracosanoic acid, as TMS ether-derivative). This series was present in the range from 2-hydroxyeicosanoic acid (C_20_) to 2-hydroxyhexacosanoic acid (C_26_), and showed a similar distribution in the rind and pith with a predominance of the even over the odd carbon atom number homologues and with 2-hydroxytetracosanoic acid (C_24_; 7) being the most abundant in both samples (110 mg/kg in the rind, and 375 mg/kg in the pith), as shown in the histograms of **Figures 6B**, **7B**. 2-Hydroxyfatty acids have also been widely found in other plants, such as hemp (Gutiérrez et al., 2006), abacá (del Río and Gutiérrez, 2006) or curauá (Marques et al., 2007), and with a similar distribution as found in papyrus. However, while only minor amounts of 2-hydroxyfatty acids (4.3 mg/kg) were found in hemp (Gutiérrez et al., 2006) and abacá (8.2 mg/kg; del Río and Gutiérrez, 2006), these compounds were found in significant quantities in the leaves of curauá (226 mg/kg; Marques et al., 2007).

Fatty alcohols were found in the range from *n*-eicosanol (C_20_) to *n*-octacosanol (C_28_), with a predominance of the even-numbered homologues, and with a similar distribution in the rind and in the pith, as shown in the histograms of **Figures 6C**, **7C**. The series of fatty alcohols accounted for a total of 280 and 65 mg/kg in rind and pith, respectively. The major compound of the series in both samples was *n*-eicosanol (C_20_; 8), accounting for 66 mg/kg in the rind and 15 mg/kg in the pith. The amounts of fatty alcohols reported in plant materials vary widely from as low as 4 mg/kg and 14 mg/kg in the pith and cortex of elephant grass, respectively (Prinsen et al., 2012), to 318 mg/kg in flax (Gutiérrez and del Río, 2003), and 552 mg/kg in the leaves of curauá (Marques et al., 2007), to as high as 1615 mg/kg in wheat straw (del Río et al., 2013).

Acylglycerols, specifically mono- and diglycerides, were found in these samples, while triglycerides could not be detected. Among the monoglycerides, the 1-monoglycerides class was the most prominent (650 mg/kg in the rind, and 830 mg/kg in the pith), while the 2-monoglycerides were found in lesser amounts (90 mg/kg in the rind, and 120 mg/kg in the pith). The isomeric monoglycerides were identified based on their characteristic mass spectra, as reflected in **Figure S2** for 1-monooctacosanyl glycerol (as its TMS-ether derivative), and in **Figure S3** for 2-monooctacosanyl glycerol (as its TMS-ether derivative). The series of 1-monoglycerides ranged from 1-monohexadecanoylglycerol (1-monopalmitin, C_16_; 9) to 1-monodotriacontanoylglycerol (C_32_), with a strong predominance of the even-carbon homologues. However, the distribution of this series was different in the rind and the pith, as shown in the histograms of **Figures 6D**, **7D**. While the rind presented a maximum for 1-monohexadecanoylglycerol (C_16_), the pith presented a higher content of high molecular weight 1-monoglycerides with a maximum for 1-monohexacosanoylglycerol (C_26_). Important amounts of unsaturated 1-monoglycerides, including 1-monooctadec-9-enoylglycerol (1-monoolein, C_18:1_), 1-monooctadec-9,12-dienoylglycerol (1-monolinolein, C_18:2_), and 1-monooctadec-9,12,15-trienoylglycerol (1-monolinolenin, C_18:3_), were also found, being more abundant in the rind. The series of 2-monoglycerides were found in the range from 2-monohexadecanoylglycerol (2-monopalmitin, C_16_; 10) to 2-monotriacontanoylglycerol (C_30_), with the exclusive occurrence of the even-carbon homologues, and maxima for 2-monooctacosanoylglycerol (C_28_) in the rind, and for 2-monohexacosanoylglycerol (C_26_) in the pith. Unsaturated 2-monoglycerides were not detected. On the other hand, diglycerides were found in both parts of the papyrus stem, being more abundant in the pith (610 mg/kg) than in the rind (270 mg/kg), and were identified by the mass spectra of their TMS-ether derivatives (Curstedt, 1974). The main compounds identified were 1,2-dipalmitin (11) and 1,3-dipalmitin (12) as well as the unsaturated 1,2- and 1,3-palmitoylolein, with a predominance of the 1,3-positional isomers. Finally, an acylglycerol glycoside, namely lyso-monogalactosyl-monopalmitin (13), was found in both parts of the papyrus stems, and was tentatively identified by comparison of its mass spectrum (as TMS-ether derivative) with that previously published (Lai et al., 2018). Lyso-monogalactosyl-monopalmitin was found to be more abundant in the pith (200 mg/kg) than in the rind (50 mg/kg).

A series of alkylamides was also found in both parts of the papyrus stems, with a predominance of the unsaturated oleic amide (C_18:1_; 14) that accounted for 185 mg/kg in the rind and 175 mg/kg in the pith. In addition, high molecular weight alkylamides ranging from triacontanamide (C_30_) to tetratriacontanamide (C_34_) were also found in the rind, with the exclusive occurrence of the even-carbon homologues and with dotriacontanamide (C_32_) as the most important member, but they were not detected in the pith. The mass spectra of representative alkylamides are shown in **Figure S4** (for oleic amide, underivatized and as its TMS-derivative) and in **Figure S5** (for dotriacontanamide, underivatized). Alkylamides have been found in a wide range of plant families. They are of interest to the pharmaceutical industries as they are claimed to possess pharmacological activities, such as antimicrobial, analgesic and anti-inflammatory properties. As such they have been commonly used in traditional medicine (Boonen et al., 2012; Veryser et al., 2016).

The unsaturated isoprenoid alcohol phytol (15) was identified in both parts of the papyrus stems, being present in higher abundance in the rind (260 mg/kg) than in the pith (20 mg/kg). Interestingly, a series of phytol fatty acid esters formed by esterification of phytol with different long-chain fatty acids were also found in important amounts in the rind (4725 mg/kg), and in lesser amounts in the pith (520 mg/kg), as occurs with free phytol. The phytol esters were identified by their characteristic mass spectra, with a base peak at *m/z* 123 and an intense mass fragment at *m/z* 278 arising from the isoprenoid chain (Krauß and Vetter, 2018). The mass spectrum of a representative member of the phytol ester series (phytyl hexadecanoate) is shown in **Figure S6**. Phytol esters have been widely identified in the wax ester fraction of several plants (Klink et al., 1994; van Bergen et al., 1997; Gaude et al., 2007; Krauß and Vetter, 2018; Krauß et al., 2018). In papyrus stems, the phytol esters series ranged from phytyl dodecanoate (C_12_) to phytyl hexacosanoate (C_26_), with a strong predominance of the even-carbon homologues, and with a maximum for phytyl hexadecanoate (C_16_; 16) in the rind (accounting for 1430 mg/kg), and for phytyl octadecanoate (C_18_) in the pith (accounting for 90 mg/kg), as shown in the histograms of **Figures 6E**, **7E**. Significant amounts of phytol esterified with unsaturated fatty acids were also detected, being more abundant in the rind. The unsaturated phytol esters were identified by their characteristic mass spectra that showed a base peak for the RCO_2_^+^ fragment (i.e. *m/z* 277 for phytyl octadec-9,12,15-trienoate, as shown in the mass spectrum of **Figure S6**), as previously published (Gaude et al., 2007; Krauß and Vetter, 2018), instead of the characteristic base peak at *m/z* 123 for the saturated homologues. Phytyl octadeca-9,12-dienoate (C_18:2_) and phytyl octadec-9,12,15-trienoate (C_18:3_) were the most prominent unsaturated phytol esters in both parts of the papyrus stems. Phytol and its derivatives are known to exhibit a wide range of bioactivities and are largely used in beverages and pharmaceuticals along with cosmetics (Islam et al., 2018).

### Ferulic acid derivatives

Different series of ferulic acid derivatives arising from ferulic acid esterified to different long-chain alcohols and ω-hydroxyfatty acids, forming series of alkyl ferulates and ω-carboxyalkyl ferulates, as well as monoglycerides of ω-carboxyalkyl ferulates, were also identified in both parts of the papyrus stem. These compounds were identified by their characteristic mass spectra, as previously published (del Río et al., 2004). The mass spectra of representative members of these series are shown in **Figure S7** (for *trans*-docosanylferulate, as its TMS-ether derivative), in **Figure S8** (for *trans*-feruloyloxydocosanoic acid, as it TMS derivative), and in **Figure S9** (for 1-mono-*trans*-feruloyloxydocosanoyl glycerol, as TMS-ether derivative).

Ferulic acid esters have been widely reported to be present in different plants, esterified either with long-chain fatty alcohols (Freire et al., 2002; del Río et al., 2004; del Río and Gutiérrez, 2006; Gutiérrez et al., 2008; Marques et al., 2010) or with ω-hydroxyfatty acids (Kawanishi and Hashimoto, 1987; del Río et al., 2004; del Río and Gutiérrez, 2006; Gutiérrez et al., 2008). Moreover, monoglycerides of ω-carboxyalkyl ferulates have also been identified in some plants (Kawanishi and Hashimoto, 1987). In papyrus stems, alkylferulates were found in significant amounts in the rind (90 mg/kg) and in the pith (85 mg/kg); similar amounts of alkylferulates (91.5 mg/kg) were found in sisal (Gutiérrez et al., 2008), while lower amounts (21.4 mg/kg) were reported in the leaves of abacá (del Río and Gutiérrez, 2006). This series ranged from octadecyl ferulate (C_18_) to octacosanyl ferulate (C_28_), as shown in the histograms of **Figures 6F**, **7F**. Only the even-numbered homologues were found, with *trans*-docosanyl ferulate (C_22_; 17) being the most prominent one in the rind (32 mg/kg) while *trans*-hexacosanyl ferulate (C_26_) was the most prominent one in the pith (28 mg/kg). Also ω-carboxyalkyl ferulates were found in significant amounts, accounting for 340 mg/kg in the rind and 325 mg/kg in the pith. Only minor amounts of ω-carboxyalkyl ferulates (2.5 mg/kg) were previously reported in both sisal (Gutiérrez et al., 2008) and abacá (del Río and Gutiérrez, 2006). This series ranged from feruloyloxyeicosanoic acid (C_20_) to feruloyloxyoctacosanoic acid (C_28_), with the occurrence of both odd- and the even-numbered homologues, although with a strong predominance of the latter, as shown in the histograms of **Figures 6G**, **7G**. As occurred with the series of alkylferulates, the most prominent member in the rind was feruloyloxydocosanoic acid (C_22_; 18; 90 mg/kg), while the most prominent members in the pith were feruloyloxytetracosanoic acid (C_24_, 80 mg/kg) and feruloyloxyhexacosanoic acid (C_26_, 80 mg/kg). Finally, a series of monoglycerides of ω-carboxyalkyl ferulates was also found among the lipids of papyrus stems, accounting for 325 mg/kg in the rind and 500 mg/kg in the pith. The distributions of this class of compounds are represented in the histograms of **Figure 6H** (for the rind) and **Figure 7H** (for the pith). Only the even-carbon homologues were found in both parts of the papyrus stems, ranging from 1-mono-*trans*-feruloyloxyeicosanoylglycerol (C_20_) to 1-mono-*trans*-feruloyloxytriacontanoylglycerol (C_30_), and with maxima for 1-mono-*trans*-feruloyloxytetracosanoylglycerol (C_24_; 19) in the rind (135 mg/kg) and for 1-mono-*trans*-feruloyloxyhexacosanoylglycerol (C_26_) in the pith (190 mg/kg).

### Tocopherols

Different tocopherols, such as α-tocopherol (20), β-tocopherol (21), γ-tocopherol (22), and δ-tocopherol (23), which are distinguished by different methylation patterns on the aromatic ring, were also identified in papyrus stems being present in higher abundance in the rind (205 mg/kg) than in the pith (45 mg/kg). Tocopherols were identified based on their characteristic mass spectra, as previously published (Snyder et al., 1993; del Río et al., 2015). In both parts of the plant, α-tocopherol was the most abundant one, accounting for 120 mg/kg in the rind and 25 mg/kg in the pith, followed by γ-tocopherol that accounted for 40 mg/kg in the rind and 10 mg/kg in the pith. The rind also contained significant amounts of δ-tocopherol (35 mg/kg) and lower amounts of β-tocopherol (10 mg/kg), whereas the pith presented only minor amounts of β-tocopherol (5 mg/kg) and δ-tocopherol (5 mg/kg).

**Figure 8:**
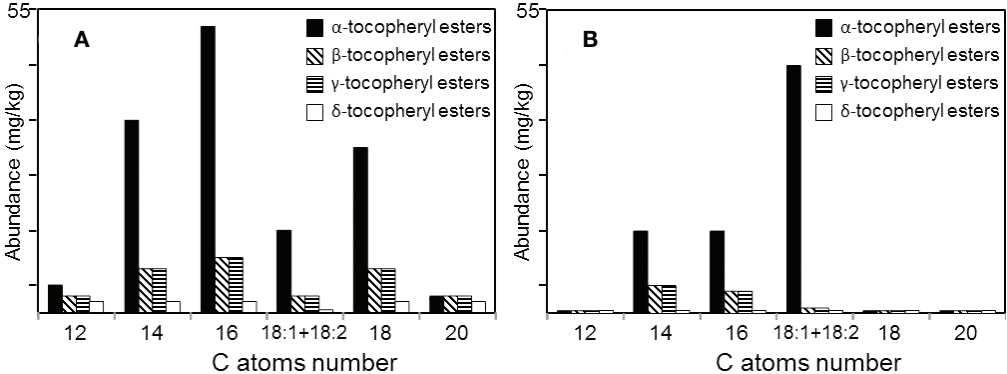
Distribution of the different series of tocopherol esters among the lipids of **(A)** the rind, and **(B)** the pith of papyrus stems.

Interestingly, different series of tocopherol esters, including esters of α-, β-, γ-, and δ-tocopherols, were also identified among the lipids of papyrus stems. The tocopherol esters were present in higher amounts in the rind (220 mg/kg) than in the pith (95 mg/kg). The tocopherol esters were identified based on their characteristic mass spectra that show a base peak corresponding to the tocopheryl fragment ion due to the loss of the fatty acid, resulting in the ions at *m/z* 430, *m/z* 416 and *m/z* 402 for the α-, β-/γ-, and δ-tocopheryl esters, respectively; these fragments undergo a retro-Diels-Alder reaction with hydrogen transfer to give an intense ion at *m/z* 165, 151 and 137 respectively, and also an α-cleavage to give a weaker ion at *m/z* 205, 191 and 177 (for the α-, β-/γ-, and δ-tocopheryl esters, respectively), as previously published (Klink et al., 1994; Rencoret et al., 2006; Kreps et al., 2017; Krauß et al., 2018). The mass spectra of representative members of the α-tocopherol esters (α-tocopheryl hexadecanoate) and of the β-tocopherol esters (β-tocopheryl hexadecanoate) are shown in **Figure S10** and in **Figure S11**, respectively. The distribution of the different tocopherol ester series in the rind and pith is shown in **Figure 8**. Similar to the free tocopherols, the α-tocopherol esters were most abundant in both parts of the papyrus stem, accounting for a total of 140 mg/kg in the rind and 75 mg/kg in the pith. In all cases, the tocopherol ester series ranged from α-, β-, γ-, and δ-tocopheryl dodecanoate (C_12_) to α-, β-, γ-, and δ-tocopheryl eicosanoate (C_20_), with the exclusive occurrence of the even-numbered homologues and with the additional presence of tocopherols esterified to the unsaturated oleic (C_18:1_) and linoleic (C_18:2_) fatty acids. In the rind, the major specimen among the α-tocopherol esters was α-tocopheryl hexadecanoate (C_16_; 24), followed by α-tocopheryl tetradecanoate (C_14_) and α-tocopheryl octadecanoate (C_18_), with lower amounts of the unsaturated α-tocopheryl oleate/linoleate (C_18:1_/C_18:2_) that eluted together. However, in the pith, the α-tocopherol ester series was dominated by the unsaturated α-tocopheryl oleate/linoleate (C_18:1_/C_18:2_), with lower amounts of α-tocopheryl tetradecanoate (C_14_) and α-tocopheryl hexadecanoate (C_16_). The β- and δ-tocopherol esters showed the same distribution in both the rind and the pith, with the predominance of the unsaturated counterparts, and with lower amounts of the unsaturated homologues. δ-Tocopherol esters were detected in the rind in low amounts from δ-tocopheryl dodecanoate (C_12_) to δ-tocopheryl eicosanoate (C_20_) and with only trace amounts of the unsaturated homologues δ-tocopheryl oleate/linoleate (C_18:1_/C_18:2_). In the pith, the δ-tocopherol ester series were only found in trace amounts. Series of α-, β-, γ-, and δ-tocopherols esterified with different long chain fatty acids in the range of C_12_ to C_20_, including the unsaturated oleic (C_18:1_) and linoleic (C_18:2_) acids have been reported to be present in the wax ester fraction of several plants (Klink et al., 1994; Rencoret et al., 2006; Krauß et al., 2018).

### Steroid compounds

Steroid compounds represent the most important group of compounds identified among the lipids of papyrus stems, accounting for 6255 mg/kg in the rind and 8600 mg/kg in the pith. Different classes of steroid compounds were found, including free sterols, steroid ketones, steroid hydrocarbons, sterol esters, sterol glycosides, and acyl sterol glycosides. The most abundant class of steroids was free sterols, which accounted for 3080 mg/kg in the rind and 5420 mg/kg in the pith, followed by sterol glycosides (1780 vs. 2140 mg/kg), steroid hydrocarbons (710 vs. 640 mg/kg), steroid ketones (320 vs. 200 mg/kg), and sterol esters (365 and 200 mg/kg in rind and pith). These are among the highest amounts of steroid compounds found in the stems of plant materials. For comparison, rice and wheat straw, two lignocellulosic materials with high abundance of steroid compounds, presented 1600 and 1121 mg/kg of sterols, respectively, 1380 and 680 mg/kg of sterol glycosides, 60 and 16 mg/kg of steroid hydrocarbons, 900 and 88 mg/kg of steroid ketones, and 380 and 70 mg/kg of sterol esters (del Río et al., 2013; Rosado et al., 2022).

Among the free sterols identified, sitosterol (25) was the most prominent in both samples, accounting for 1720 mg/kg in the rind and 2280 mg/kg in the pith. Other sterols that were present in significant amounts were campesterol (26; 370 and 1040 mg/kg in the rind and pith), and stigmasterol (27; 620 and 1350 mg/kg in the rind and pith). Other sterols present in lower amounts included campestanol (28), stigmastanol (29), Δ^5^-avenasterol (30), Δ^7^-stigmastenol (31), 7-oxocampesterol (32), 7-oxo-stigmasterol (33), 7-oxositosterol (34), ergostane-3,5,6-triol (35), and sitostane-3,5,6-triol (36), which were found in higher amounts in the pith than in the rind (**Table 1**).

Steroid ketones were dominated by stigmasta-3,5-dien-7-one (37), which accounted for 110 mg/kg in the rind and 50 mg/kg in the pith. Other steroid ketones identified in both parts of papyrus stems were stigmast-4-en-3-one (38), stigmasta-4,6-dien-3-one (39), and stigmatane-3,6-dione (40), as well as the respective ergostane-skeleton homologues, such as ergosta-3,5-dien-7-one (41), ergost-4-en-3-one (42), ergosta-4,6-dien-3-one (43), and ergostane-3,6-dione (44).

Steroid hydrocarbons were also identified, with stigmasta-3,5,22-triene (45) being the most abundant steroid hydrocarbon in both parts of papyrus stem (430 mg/kg in the rind, and 340 mg/kg in the pith). Other steroid hydrocarbons identified were stigmasta-3,5-diene, as well as the respective ergostane-counterparts ergosta-3,5,22-triene and ergosta-3,5-diene.

**Figure 9:**
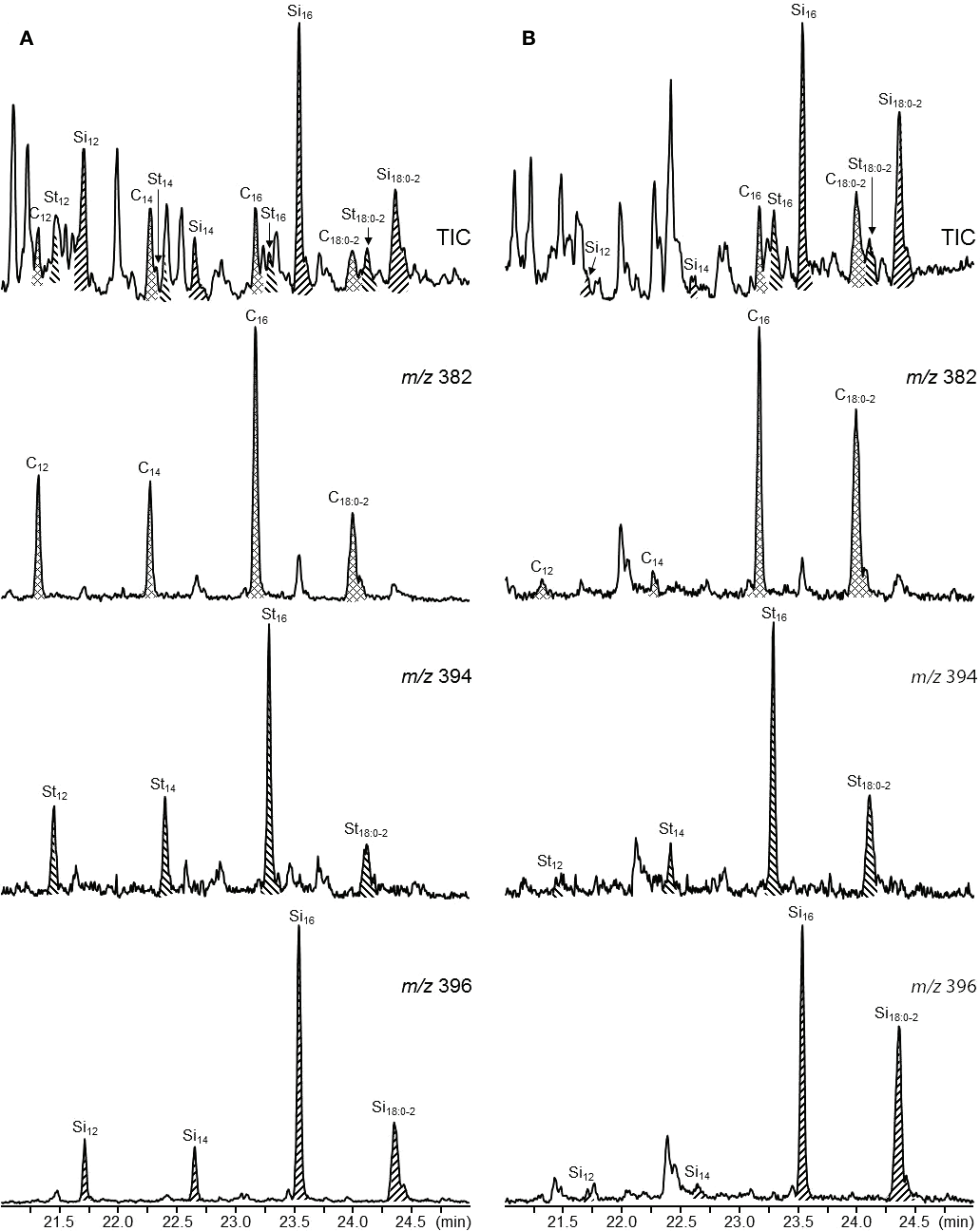
Total Ion Chromatograms (TIC) and single ion chromatograms showing the distribution of the different sterol ester series in **(A)** the rind, and **(B)** the pith of papyrus stems (*m/z* 382, campesterol esters; *m/z* 394, stigmasterol esters; *m/z* 396, sitosterol esters). C, campesterol esters; St, stigmasterol esters; Si, sitosterol esters.

Series of sterol esters were also identified in both parts of the papyrus stem, and included esters of campesterol, stigmasterol, and sitosterol. Their structures were determined by their mass spectra that showed a strong base peak corresponding to the steroid moiety (Evershed et al., 1989). **Figure 9** shows the distribution of the different series of sterol esters by monitoring the characteristic fragments of the different sterol moieties in their mass spectra. Hence, the series of sterol esters identified in papyrus stems corresponded to campesterol (*m/z* 382), stigmasterol (*m/z* 394), and sitosterol (*m/z* 396) esterified to different fatty acids, ranging from dodecanoic (C_12_) to octadecanoic acid (C_18_), including the unsaturated oleic (C_18:1_) and linoleic (C_18:2_) acids. Sitosterol esters were the most abundant sterol esters, with sitosteryl hexadecanoate (C_16_; 46) being the most prominent compound (120 mg/kg in the rind, and 45 mg/kg in the pith).

Finally, sterol glycosides and acyl sterol glycosides were also found to be present in significant amounts in both parts of the papyrus stem, accounting for 1780 mg/kg in the rind and 2140 mg/kg in the pith. These sterol conjugates widely occur in plants (Kovganko and Kashkan, 1999; Schrick et al., 2012), and were unambiguously identified as their TMS-ether derivatives by their characteristic mass spectra and by comparison with authentic standards (Gutiérrez and del Río, 2001). The most abundant sterol glycoside was sitosteryl 3-*O*-β-d-glucopyranoside (47) that amounted up to 990 mg/kg in the rind and 1210 mg/kg in the pith, followed by lesser amounts of campesteryl 3-*O*-β-d-glucopyranoside and stigmasteryl 3-*O*-β-d-glucopyranoside. Minor amounts of 7-oxo-sterol glycosides were also identified, including 7-oxo-campesteryl 3-*O*-β-d-glucopyranoside, 7-oxo-stigmasteryl 3-*O*-β-d-glucopyranoside, and 7-oxo-sitosteryl 3-*O*-β-d-glucopyranoside (**Table 1**). In addition to these sterol glycosides, a number of acyl sterol glycosides were also found in significant amounts in both parts of the papyrus stem. Acylation with different fatty acids occurred at the OH-6 of the glucopyranose moiety. The identification of the acyl group was based on the characteristic fragmentation pattern in the mass spectra corresponding to the loss of the sterol moiety (producing the fragment ions at *m/z* 617 for palmitic acid, at *m/z* 645 for stearic acid, at *m/z* 643 for oleic acid, at *m/z* 641 for linoleic acid, and at *m/z* 639 for linolenic acid), with subsequent loss of trimethylsilanol, producing the fragment ions at *m/z* 527 for palmitic acid, *m/z* 555 for stearic acid, *m/z* 553 for oleic acid, *m/z* 551 for linoleic acid, and *m/z* 549 for linolenic acid, as previously described (Gutiérrez and del Río, 2001). The acyl sterol glycosides was dominated by sitosteryl (6′-*O*-palmitoyl)-3-*O*-β-d-glucopyranoside (48), that amounted to 200 mg/kg in the rind and 110 mg/kg in the pith, and also included lower amounts of the structurally related campesteryl (6′-*O*-palmitoyl)-3-*O*-β-d-glucopyranoside, and stigmasteryl (6′-*O*-palmitoyl)-3-*O*-β-d-glucopyranoside. Likewise, campesteryl, stigmasteryl and sitosteryl 3-*O*-β-d-glucopyranosides were also acylated with a mixture of different C_18_ fatty acids (stearic, oleic, linoleic and linolenic acids), which co-eluted in the same chromatographic peak, with sitosterol (as a mixture of 6′-*O*-stearoyl/oleyl/linoleyl/linolenyl)-3-*O*-β-d-glucopyranoside) being most abundant in both parts of the papyrus stem.

The wide variability and significant amounts of steroid compounds present in papyrus stems is of great interest since these compounds are well known for their nutraceutical and health-promoting benefits, such as helping to reduce blood cholesterol levels, among others (Wilson et al., 2000; Moreau et al., 2002; Quílez et al., 2003), so it can be regarded as a useful source for obtaining these valuable phytochemicals.

## Conclusions

Papyrus stems have a significant content of lipophilic compounds that can potentially be extracted and valorized. Therefore, the detailed content and chemical composition of the lipophilic compounds in the two distinct anatomical parts of papyrus stems, that is the rind and pith, have been comprehensively studied. A wide range of acetone-extractable lipophilic compounds were found in these two parts of papyrus stems, including hydrocarbons, fatty acids, 2-hydroxyfatty acids, fatty alcohols, phytol and phytol esters, alkylamides, mono- and diglycerides, steroids (sterols, ketones, hydrocarbons, esters and glycosides), tocopherols, tocopherol esters, alkyl ferulates, ω-carboxyalkyl ferulates (and their monoglycerides), and acylglycerol glycosides. These compounds can be very useful for the pharmaceutical, nutraceutical, and chemical industries as, for example, it has already been implemented for phytol and its derivatives, as well as for alkylamides, steroids and tocopherols, which are known for exhibiting a wide range of bioactivities and are largely used in beverages and pharmaceuticals along with cosmetics. The extraction and valorization of the lipid components of papyrus will provide additional value and will promote the use of papyrus as an interesting feedstock in future lignocellulosic biorefineries.

## Data availability statement

The raw data supporting the conclusions of this article will be made available by the authors, without undue reservation.

## Author contributions

FB, TR, and AP collected and prepared the samples. MR and GM made the experimental work. AG and JR contributed to method development. JCdR designed the work, processed the data, and wrote the article, with contributions from the rests of authors. All authors approved the version submitted.
