# Supplementary material for: Chemical composition of the lipophilic compounds from the rind and pith of papyrus (Cyperus papyrus L.) stems
Source: Front Plant Sci. 2022 Dec 22;13:1097866. doi: 10.3389/fpls.2022.1097866 (PMC9813494; doi:10.3389/fpls.2022.1097866)
Supplement: Supplementary file 1 [file DataSheet_1.pdf]

Supplementary Material

Chemical Composition of the Lipophilic Compounds from the Rind and Pith of Papyrus (*Cyperus papyrus*) Stems

Mario J. Rosado,[1] Gisela Marques,[1] Jorge Rencoret,[1] Ana Gutiérrez,[1] Florian Bausch,[2] Thomas Rosenau,[2] Antje Potthast,[2] and José C. del Río[1*]

[1]Instituto de Recursos Naturales y Agrobiología de Sevilla, CSIC, Avda. Reina Mercedes, 10, 41012-Seville, Spain

[2]Institute of Chemistry of Renewable Resources, University of Natural Resources and Life Sciences Vienna (BOKU), Konrad-Lorenz-Straße 24, A-3430 Tulln, Austria

*Corresponding author: delrio@irnase.csic.es

Supplementary Figures

Figure S1. Mass spectrum of 2-hydroxytetracosanoic acid (as TMS-ether derivative).

Figure S2. Mass spectrum of 1-monooctacosanyl glycerol (as TMS-ether derivative).

Figure S3. Mass spectrum of 2-monooctacosanyl glycerol (as TMS-ether derivative).

Figure S4. Mass spectrum of oleic amide, (A) underivatized, and (B) as TMS-ether derivative.

Figure S5. Mass spectrum of dotriacontanamide.

Figure S6. Mass spectrum of (A) phytyl hexadecanoate, and (B) phytyl octadeca-9,12,15-trienoate.

Figure S7. Mass spectrum of *trans*-docosanylferulate (as TMS-ether derivative).

Figure S8. Mass spectrum of *trans*-feruloyloxydocosanoic acid (as TMS-ether derivative).

Figure S9. Mass spectrum of 1-mono-*trans*-feruloyloxydocosanoyl glycerol (as TMS-ether derivative).

Figure S10. Mass spectrum of α-tocopheryl hexadecanoate.

Figure S11. Mass spectrum of β-tocopheryl hexadecanoate.

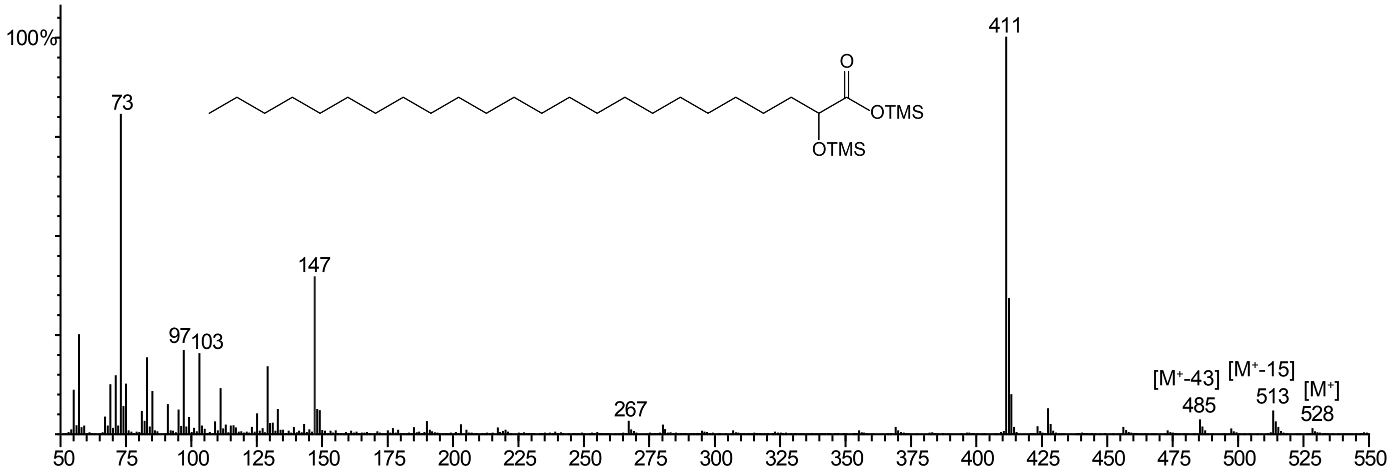

Figure S1. Mass spectrum of 2-hydroxytetracosanoic acid (as TMS-ether derivative).

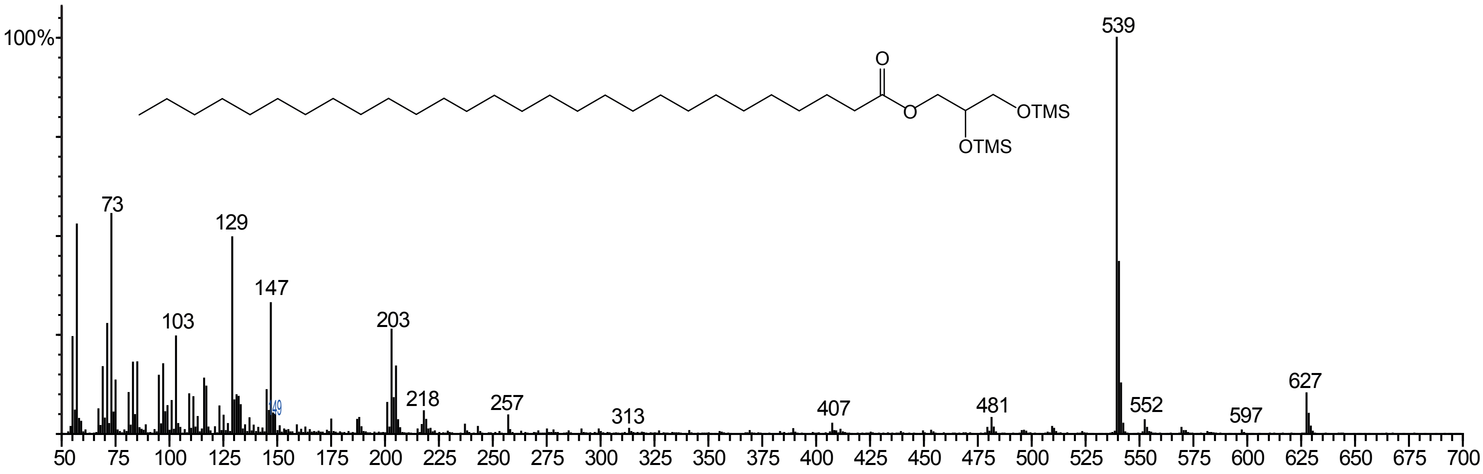

Figure S2. Mass spectrum of 1-monooctacosanyl glycerol (as TMS-ether derivative).

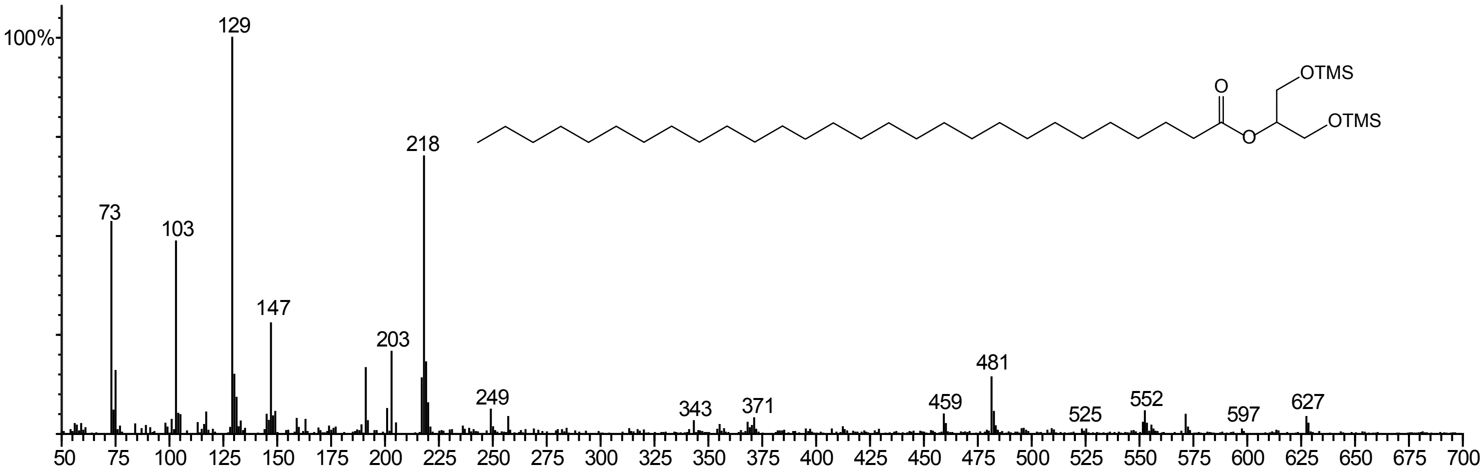

Figure S3. Mass spectrum of 2-monooctacosanyl glycerol (as TMS-ether derivative).

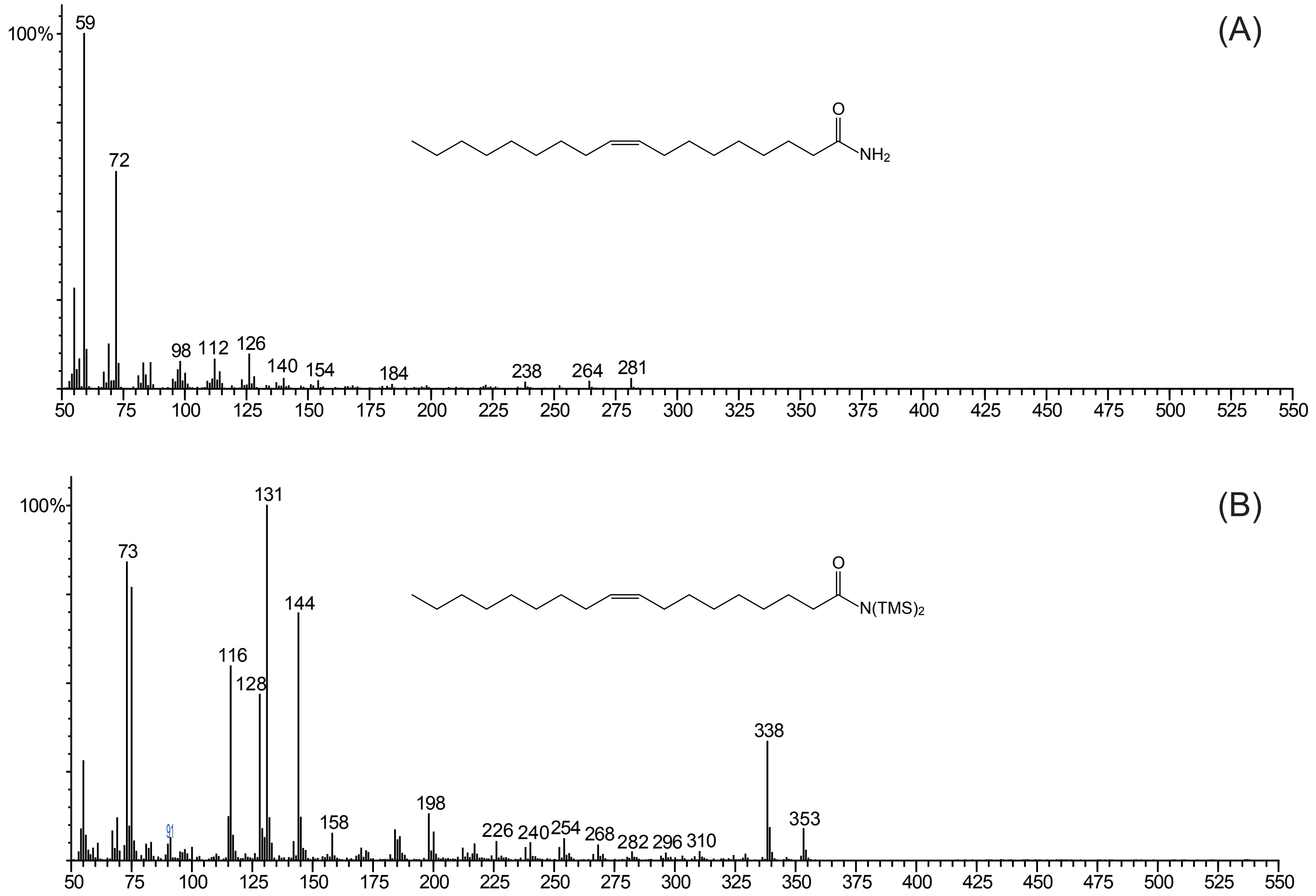

Figure S4. Mass spectrum of oleic amide, (A) underivatized, and (B) as TMS-ether derivative.

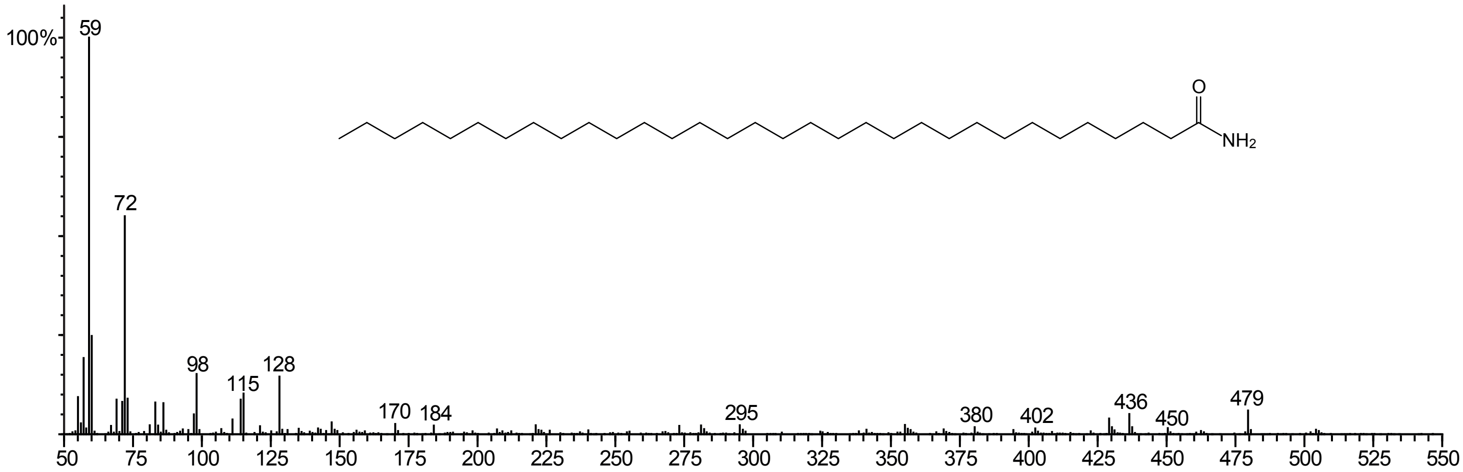

Figure S5. Mass spectrum of dotriacontanamide.

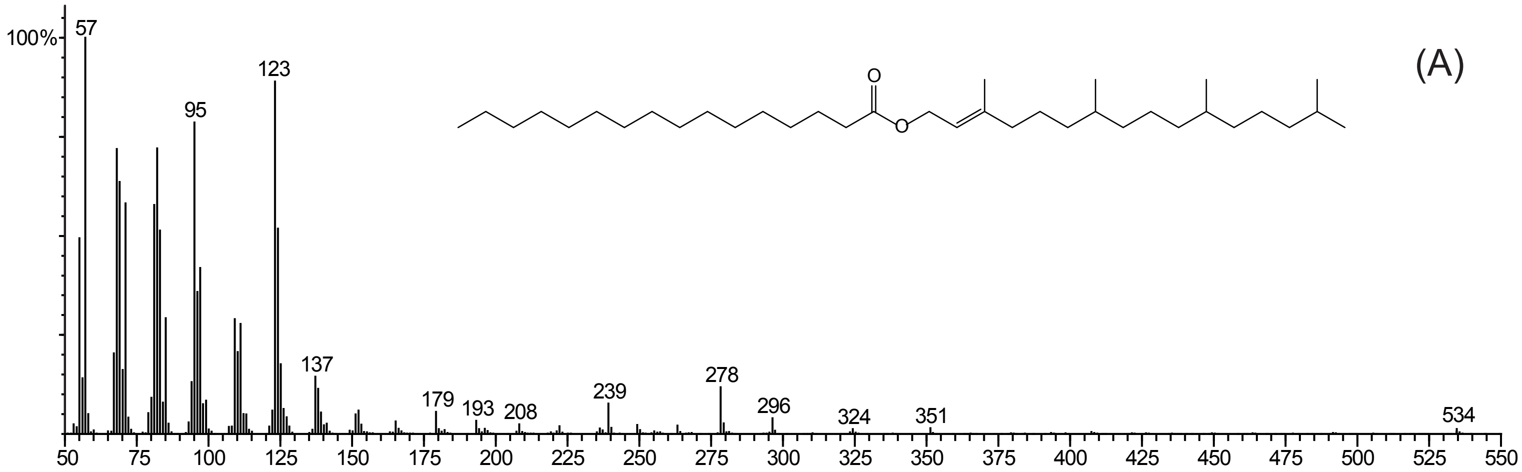

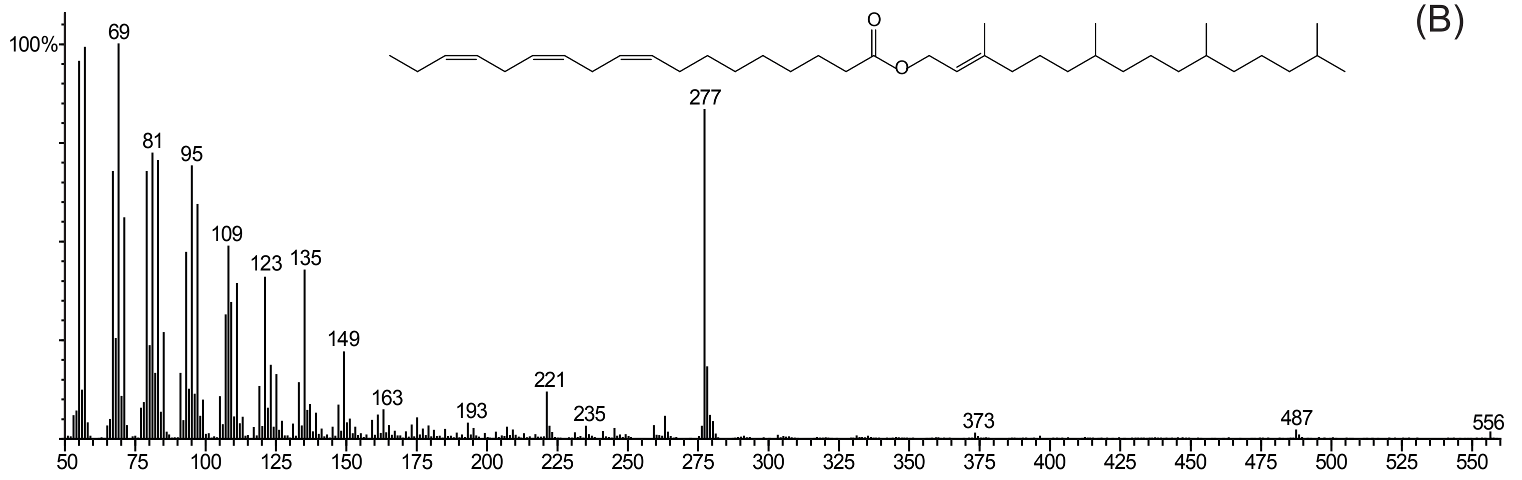

Figure S6. Mass spectrum of (A) phytyl hexadecanoate, and (B) phytyl octadeca-9,12,15-trienoate.

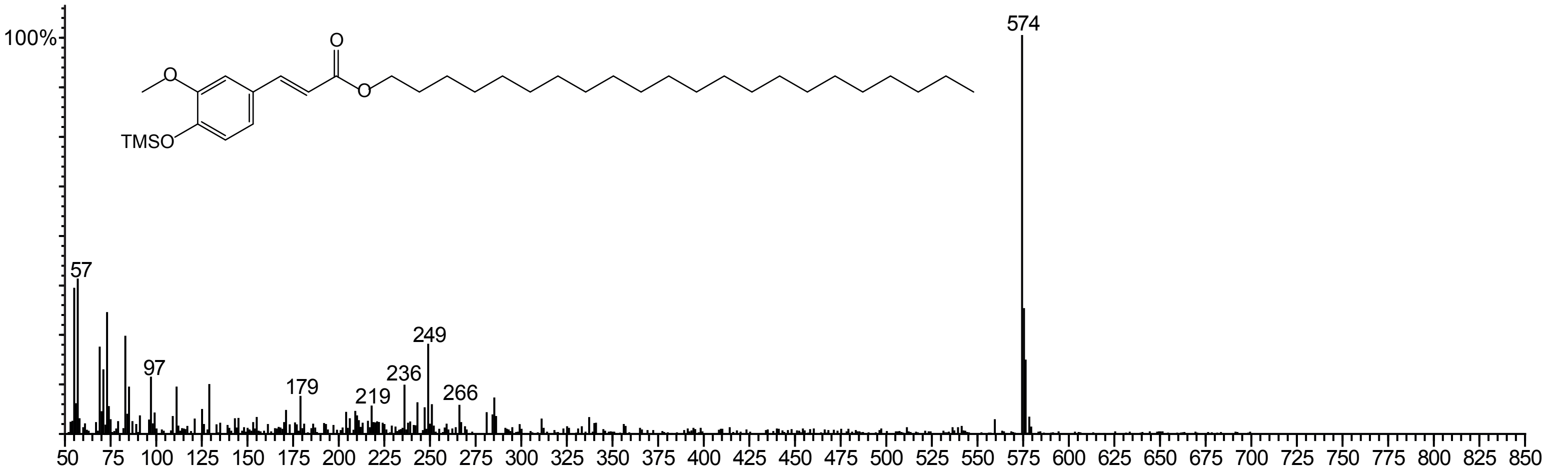

Figure S7. Mass spectrum of *trans*-docosanylferulate (as TMS-ether derivative).

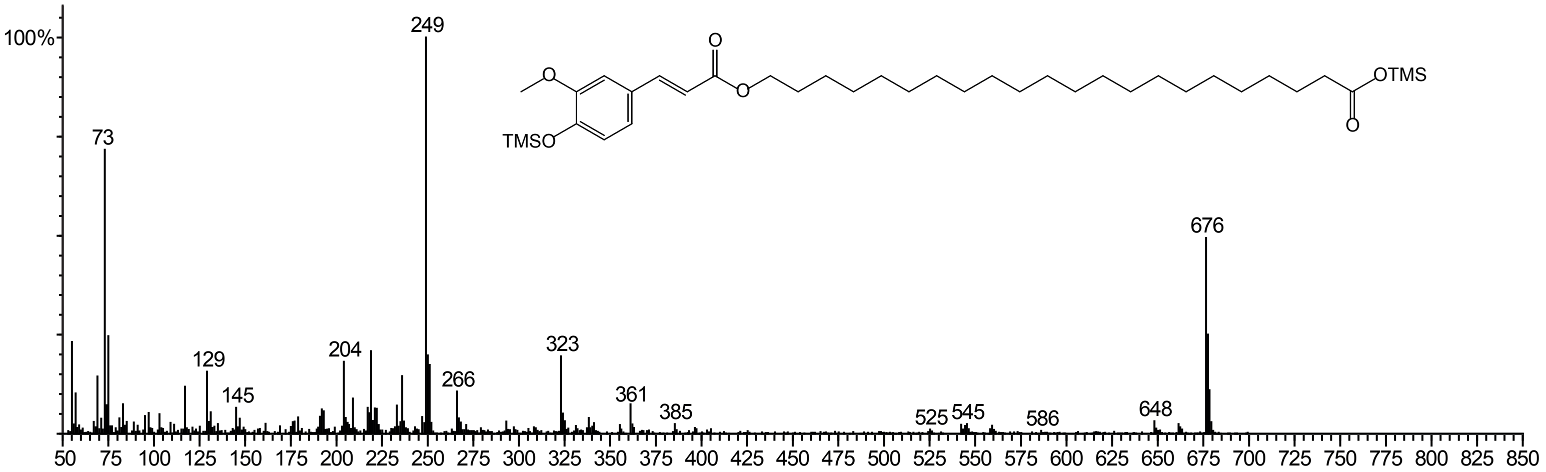

Figure S8. Mass spectrum of *trans*-feruloyloxydocosanoic acid (as TMS-ether derivative).

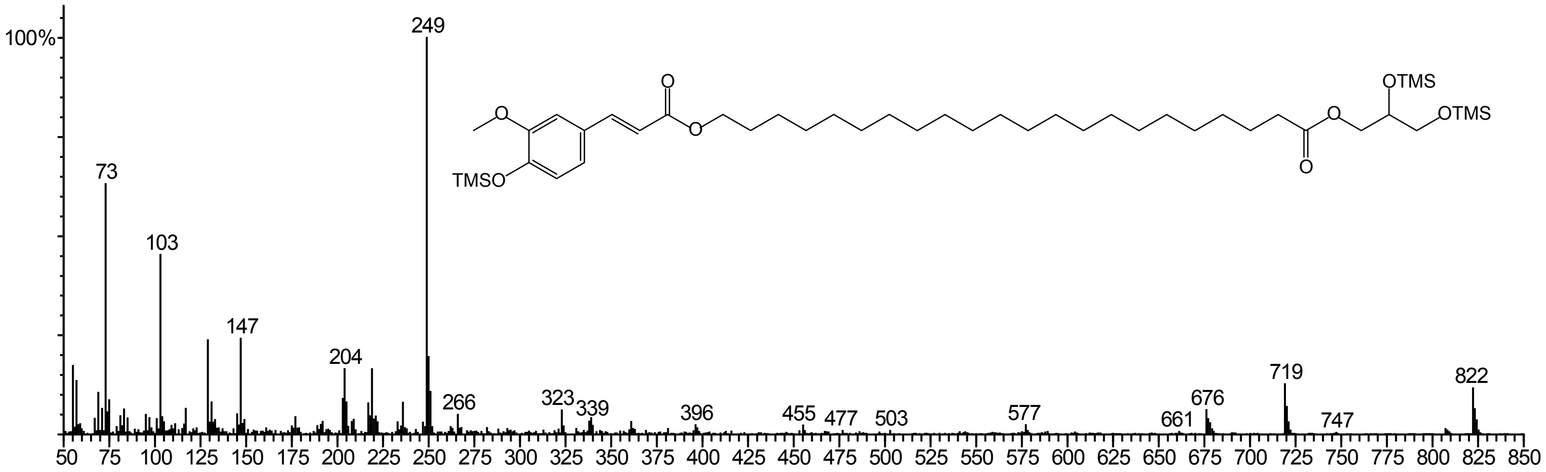

Figure S9. Mass spectrum of 1-mono-*trans*-feruloyloxydocosanoyl glycerol (as TMS-ether derivative).

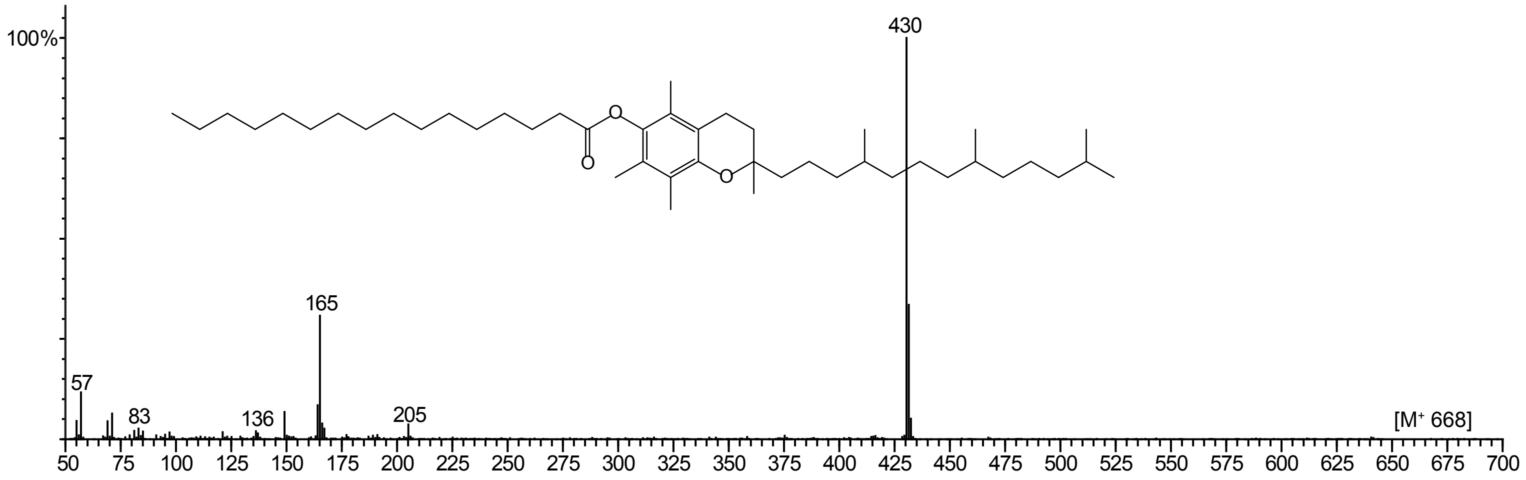

Figure S10. Mass spectrum of α-tocopheryl hexadecanoate.

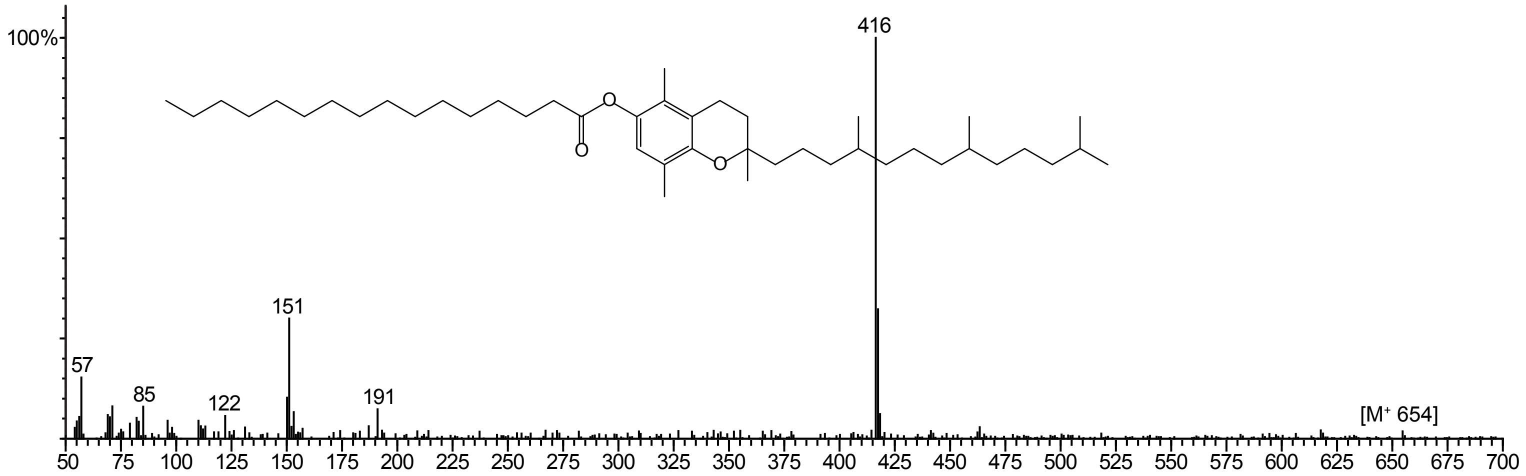

Figure S11. Mass spectrum of β-tocopheryl hexadecanoate.
